# Component network meta-analysis of scaffold materials, platelet-rich fibrin and barrier membranes for alveolar ridge preservation

**DOI:** 10.3389/froh.2026.1949538

**Published:** 2026-09-18

**Authors:** Jian Zhang, Jie Zhong, Shihan Huang, Ling Zhang

**Affiliations:** The Shengzhou Yuexin Dental Clinic, Shaoxing, Zhejiang Province, China

**Keywords:** alveolar ridge preservation, barrier membrane, component network meta-analysis, platelet-rich fibrin, scaffold material

## Abstract

**Background:**

Alveolar ridge preservation (ARP) involves the use of multiple treatment components, including scaffold materials, platelet-rich fibrin (PRF) adjuncts, and barrier membranes. However, conventional meta-analyses generally evaluate complete treatment protocols and cannot determine the independent contribution of individual components. This study aimed to investigate the component-level effects of ARP interventions using component network meta-analysis (CNMA).

**Methods:**

Randomized controlled trials evaluating ARP after tooth extraction in adults were identified from PubMed, Cochrane CENTRAL, Embase, and Web of Science from inception to July 2026. Complete treatment strategies were initially compared using random-effects network meta-analysis. Subsequently, interventions were decomposed into scaffold, PRF, and barrier membrane components for additive and interaction CNMA. The primary outcomes were horizontal ridge-width change and buccal vertical ridge-height change.

**Results:**

Thirty-one randomized controlled trials were included. Standard network meta-analysis demonstrated that several scaffold- and membrane-containing strategies were associated with reduced ridge dimensional loss compared with natural healing. In additive CNMA for horizontal ridge-width change, barrier membrane showed the largest estimated component effect (MD, −1.31 mm; 95% CI, −2.18 to −0.44), followed by allograft (MD, −0.77 mm; 95% CI, −1.16 to −0.38) and PRF adjunct (MD, −0.40 mm; 95% CI, −0.66 to −0.14). Interaction analyses did not demonstrate convincing evidence that the effect of PRF varied according to scaffold type, although estimates were limited by imprecision.

**Conclusions:**

This component network meta-analysis suggests that the effects of alveolar ridge preservation are associated with the combined contribution of individual treatment components rather than complete treatment packages alone. Barrier membranes and allograft components showed relatively favorable component-level estimates for horizontal ridge-width preservation, whereas PRF showed a smaller incremental effect. However, these findings should not be interpreted as definitive evidence of superiority over other regenerative components, given network inconsistency, sparse evidence for some components, and the low or very low certainty of several component-level estimates.

**Systematic Review Registration**: identifier CRD420261459356.

## 1 Introduction

Tooth extraction initiates a remodeling process that can lead to substantial dimensional changes of the alveolar ridge, particularly during the early stages of healing (1). Horizontal ridge reduction and buccal plate resorption may compromise prosthetically driven implant placement, increase the need for additional augmentation procedures, and affect esthetic outcomes (2). Alveolar ridge preservation (ARP) has therefore been widely adopted to minimize post-extraction dimensional alterations and maintain favorable conditions for subsequent implant rehabilitation (3, 4). However, ARP is not a single therapeutic procedure, and the most effective combination of biomaterials and adjunctive approaches remains unclear.

Current ARP protocols involve a variety of materials and techniques, including xenogeneic, allogeneic, alloplastic, collagen-based, and tooth-derived scaffolds, as well as biologic adjuncts such as platelet-rich fibrin (PRF) and different barrier or socket-sealing strategies (4, 5). In clinical practice, these approaches are frequently combined rather than applied independently. For example, a graft material may be used together with PRF or a membrane to enhance space maintenance, wound stability, or tissue regeneration (6). As a result, differences observed between ARP protocols may reflect not only the choice of a specific product but also the underlying combination of treatment components.

Previous systematic reviews and meta-analyses have investigated the effectiveness of ARP interventions; however, the relative contribution of individual treatment components remains incompletely understood (5–7). Pairwise meta-analysis can provide estimates for specific head-to-head comparisons, whereas standard network meta-analysis allows comparison of multiple complete treatment strategies using direct and indirect evidence (8). Nevertheless, these approaches generally evaluate each protocol as an integrated intervention and cannot determine whether observed effects are primarily attributable to the scaffold material, PRF adjunct, barrier membrane, or their combination. This limitation is clinically relevant because treatment decisions in ARP are typically based on selecting and combining multiple components rather than choosing a single intervention. Component network meta-analysis (CNMA) offers an alternative framework by separating complex interventions into predefined components and estimating their individual contributions while accounting for the structure of the treatment network (9, 10). Given the multifactorial nature of ARP protocols, CNMA may provide additional insight into which components are most consistently associated with ridge preservation and whether potential interactions exist between different treatment elements.

Therefore, this study aimed to evaluate the comparative effectiveness of scaffold materials, PRF adjuncts, and barrier membranes for alveolar ridge preservation after tooth extraction using evidence from randomized controlled trials. Standard network meta-analysis was conducted to compare complete ARP strategies, while CNMA was applied to estimate component-level effects and explore potential interactions between treatment components.

## 2 Materials and methods

### 2.1 Protocol and reporting

This systematic review and component network meta-analysis was conducted according to PRISMA and the PRISMA extension for network meta-analysis (11, 12). The protocol was prospectively registered in PROSPERO before formal data extraction (CRD420261459356).

### 2.2 Research question and eligibility criteria

The primary research question was formulated as follows: “Among adults undergoing permanent tooth extraction followed by alveolar ridge preservation, what are the comparative effects of different scaffold materials, platelet-rich fibrin (PRF) adjuncts, and barrier membrane strategies on alveolar ridge dimensional preservation and related clinical outcomes?”

The eligibility criteria were defined according to the PICOS framework. Population (P): Adults (≥18 years) requiring extraction of permanent teeth and subsequent alveolar ridge preservation. Studies involving third molar extraction alone were excluded unless the reported outcomes were directly relevant to implant-site preservation. Intervention (I): ARP strategies involving scaffold materials (including xenografts, allografts, alloplasts, collagen-based materials, and tooth/dentin-derived grafts), PRF-based biological adjuncts, barrier membranes, or combinations of these components. Comparator (C): Eligible comparators included spontaneous healing/blood clot healing, PRF alone, scaffold-based approaches, membrane-assisted approaches, or other ARP protocols. Outcomes (O): The primary outcomes were horizontal ridge-width change and buccal vertical ridge-height change after ARP. Secondary outcomes included histomorphometric parameters, implant-related outcomes, patient-reported outcomes, and treatment-related adverse events. Study design (S): Only randomized controlled trials (RCTs), including parallel-group, split-mouth, and multi-arm designs, were eligible.

The exclusion criteria were as follows: (1) non-randomized studies, observational studies, case reports, case series, reviews, editorials, and conference abstracts without sufficient extractable data; (2) animal or *in vitro* studies; (3) studies evaluating extraction procedures unrelated to ARP, such as immediate implant placement without a separate ARP intervention; (4) studies focusing exclusively on third molar extraction without relevance to implant-site preservation; (5) studies without sufficient information for intervention classification, quantitative synthesis, or component coding.

### 2.3 Information sources and study selection

A comprehensive literature search was conducted in PubMed, Embase, Cochrane Central Register of Controlled Trials (CENTRAL), and Web of Science from database inception to July 2026. The search strategy was developed using controlled vocabulary and free-text terms related to alveolar ridge preservation, socket preservation, platelet-rich fibrin (PRF), graft materials, barrier membranes, and randomized controlled trials. Detailed search strategies for each database are provided in Supplementary Tables S1, S2.

All retrieved records were imported into a reference management system, and duplicates were removed before screening. Two reviewers independently screened titles and abstracts, followed by full-text assessment according to the prespecified eligibility criteria. Any disagreement between reviewers was resolved through discussion, with adjudication by a third reviewer when consensus could not be reached. The reasons for full-text exclusion were documented, and the overall selection process was presented using PRISMA flow diagram (Figure 1) (11).

**Figure 1:**
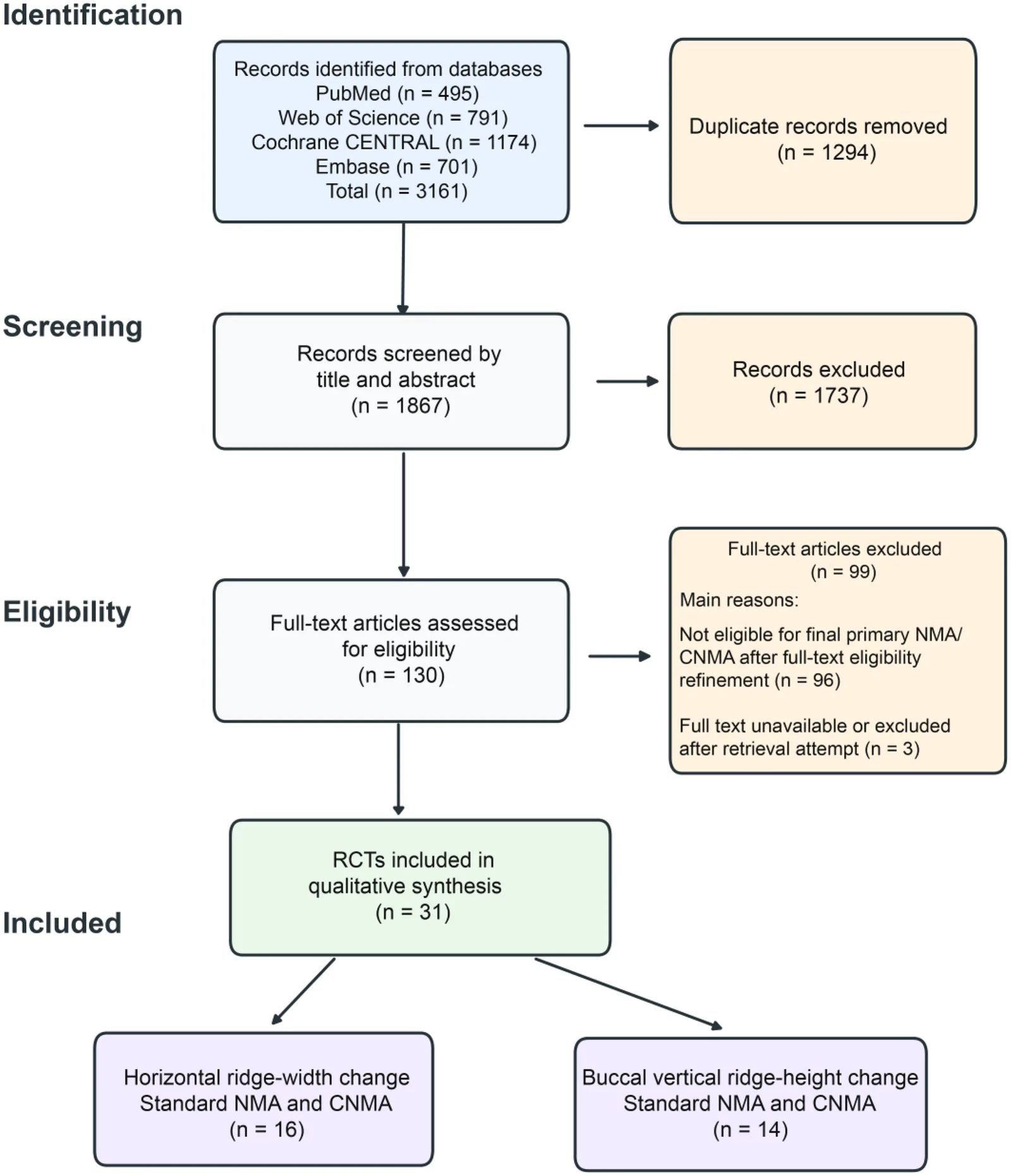
PRISMA flow diagram of study selection.

### 2.4 Data extraction and component coding

Two reviewers independently extracted data using a standardized and pilot-tested extraction form. Extracted information included study characteristics (first author, publication year, country and study design), participant and socket characteristics, intervention and comparator details, PRF preparation protocols, membrane characteristics, follow-up duration, outcome definitions, and numerical outcome data. For multi-arm trials, data from each eligible intervention arm were extracted separately. For split-mouth trials, the paired design and unit of analysis were recorded to account for potential dependence between treatment sites. When multiple reports originated from the same trial, they were considered as a single study and the most complete report was used as the primary data source.

Interventions were coded at two levels. First, complete treatment strategies were classified as treatment nodes for the standard network meta-analysis. Second, each intervention was decomposed into predefined components for component network meta-analysis (CNMA). The prespecified component framework included xenograft, allograft, alloplast, collagen-based scaffold, tooth/dentin-derived scaffold, PRF adjunct, and barrier membrane. In the primary CNMA, different PRF preparations, including L-PRF, A-PRF, injectable PRF, titanium-prepared PRF and related variants, were grouped as a single PRF adjunct component because of their shared biological rationale and limited comparative evidence. Alternative PRF coding strategies were evaluated in sensitivity analyses when data availability permitted. To assess the separability of component effects, we examined the distribution of each component across the treatment network, including its occurrence as a standalone intervention and in combination with other components.

### 2.5 Outcomes and risk of bias

The primary outcomes were changes in horizontal ridge width and buccal vertical ridge height following tooth extraction, measured in millimeters. For dimensional outcomes, greater preservation was defined as a smaller reduction in ridge dimensions compared with baseline or natural healing. Secondary outcomes included changes in lingual or palatal vertical ridge height, histomorphometric parameters (including newly formed bone, residual graft material and non-mineralized tissue), requirement for additional augmentation, implant placement feasibility, patient-reported outcomes and adverse events. Risk of bias was assessed independently by two reviewers using the Cochrane Risk of Bias 2 (RoB 2) tool (13). The assessment covered the predefined RoB 2 domains, with additional consideration given to methodological issues specific to split-mouth trials, including appropriate handling of paired data, potential carry-across effects and the independence of extraction sites. When multiple follow-up assessments were available, a single prespecified time point closest to the typical implant-site evaluation period was selected for the primary analysis to avoid multiple inclusion of data from the same study. A 4–6-month follow-up window was prespecified for sensitivity analysis to reduce clinical heterogeneity related to differences in healing and remodeling periods and because this interval commonly corresponds to clinical reassessment of ridge dimensions and implant-site readiness after ARP. For consistency, the measured outcomes were termed “horizontal ridge-width change” and “buccal vertical ridge-height change,” whereas “ridge-dimensional loss” referred to the broader post-extraction dimensional reduction process.

### 2.6 Statistical analysis

Direct comparisons supported by at least two studies were initially synthesized using random-effects pairwise meta-analysis. Standard random-effects network meta-analysis (NMA) was subsequently performed using complete intervention strategies as treatment nodes, allowing estimation of relative effects among all connected interventions through direct and indirect evidence (8, 15). Continuous outcomes were summarized as mean differences (MDs) with 95% confidence intervals (CIs). For parallel-group trials, the participant or extraction site was used as the analytical unit according to the design and reporting of the original study. For split-mouth trials, the paired design and reported unit of analysis were recorded during data extraction. When paired effect estimates or within-participant correlation coefficients were unavailable, arm-level data were analyzed as reported; the resulting potential unit-of-analysis limitation was considered in the interpretation of the findings. For multi-arm trials, correlations among comparisons arising from the same study were accounted for within the NMA framework to avoid double counting of shared intervention or comparator groups. For dimensional outcomes, effect directions were harmonized such that negative MDs indicated less dimensional loss and therefore favored better ridge preservation. Network geometry was examined using network plots, while between-study heterogeneity and network inconsistency were assessed using established statistical approaches. For component network meta-analysis (CNMA) (9, 10), complete interventions were decomposed into predefined components, including scaffold materials, platelet-rich fibrin (PRF) adjuncts and barrier membranes. Additive CNMA was used to estimate the independent contribution of each component, assuming that the overall effect of a combined intervention could be represented by the additive effects of its constituent components. The additive CNMA model assumes that the relative effect of a complete intervention can be approximated by the sum of its predefined component effects in the absence of specified interactions. Component exchangeability assumes that a given component contributes a sufficiently comparable underlying effect across the treatment combinations in which it occurs. Transitivity was considered by examining potential effect modifiers across treatment comparisons, including follow-up duration, socket morphology, tooth location, measurement method, PRF preparation, and membrane use. Consistency between direct and indirect evidence was evaluated using global and local inconsistency assessments. Given the heterogeneity of materials and techniques within several component categories, component-level estimates were interpreted as average effects within the observed evidence network rather than as product-specific effects.

Prespecified interaction models were further explored to assess potential effect modification between PRF and scaffold or membrane components when sufficient evidence was available. The standard NMA, additive CNMA and interaction CNMA models were compared using approximate AIC, residual deviance, between-study heterogeneity, and model complexity. A lower approximate AIC was interpreted as indicating a more favorable balance between model fit and parsimony rather than uniformly better absolute model fit. All analyses were performed in R software using the netmeta and netcomb packages (14).

### 2.7 Sensitivity analyses and certainty assessment

Sensitivity analyses were conducted to evaluate the robustness of the findings under different analytical assumptions. These analyses included exclusion of studies at high risk of bias, restriction to the prespecified 4–6-month follow-up window, alternative coding strategies for PRF-related interventions, and alternative classifications of scaffold materials. For the primary dimensional outcomes, potential publication bias and small-study effects were assessed descriptively using comparison-adjusted funnel plots where applicable. Formal statistical tests for funnel-plot asymmetry were not performed because several treatment nodes and direct comparisons were informed by a limited number of studies, making such tests unreliable. The certainty of evidence for treatment effects derived from the standard network meta-analysis was assessed using CINeMA (16, 17), incorporating within-study bias, reporting bias, indirectness, imprecision, heterogeneity and incoherence. For CNMA estimates, certainty was assessed using a structured narrative approach because formal grading methods for component-level effects are not yet fully standardized. The assessment considered the availability and distribution of supporting evidence, component identifiability within the network, precision of the estimate, heterogeneity and inconsistency, model dependence, and risk of bias of the contributing studies. Lower certainty judgments were generally associated with sparse or unevenly distributed evidence, imprecision, limited component identifiability, sensitivity to model specification, or important heterogeneity or inconsistency within the underlying network.

## 3 Results

### 3.1 Study selection

The database search identified 3,161 records, of which 1,294 duplicates were removed. After title and abstract screening, 130 articles were retrieved for full-text assessment. Following application of the prespecified eligibility criteria, 31 randomized controlled trials were included in the qualitative synthesis. Of these, 16 studies provided eligible data for horizontal ridge-width change and were included in the corresponding standard NMA and CNMA, whereas 14 studies provided eligible data for buccal vertical ridge-height change and were included in the corresponding standard NMA and CNMA. The remaining studies were retained in the qualitative synthesis but did not contribute to the primary quantitative networks because they did not report the relevant dimensional outcomes, provided insufficient numerical data for meta-analysis, reported outcomes that could not be harmonized with the prespecified definitions, or reported only secondary outcomes. The study selection process is summarized in the PRISMA 2020 flow diagram (Figure 1).

### 3.2 Study characteristics

The 31 included randomized controlled trials were published between 2008 and 2026 and were conducted across 17 countries or regions (18–48). The sample sizes ranged from 8 to 88 participants, with a total of 932 participants and 853 extraction sites reported across the included studies. Most trials adopted parallel-group designs, while seven used split-mouth designs and five included three or more intervention arms. Follow-up durations ranged from approximately 8 weeks to 7 months, with the majority of ridge dimensional and histomorphometric assessments performed within 3–6 months after tooth extraction. The characteristics of the included studies are summarized in Table 1, and detailed study-level extraction data are provided in Supplementary Tables S3, S4.

**Table 1:** Basic characteristics of included randomized controlled trials.

Study	Country	Design	Participants/sites	Intervention nodes	Follow-up	Outcome assessment
Estrin et al. (18)	USA	Parallel RCT, 5 arms	55/55	Sticky bone + e-PRF membrane vs. sticky bone + collagen membrane	3 mo	CBCT; clinical
Alrayyes et al. (19)	Saudi Arabia	RCT, 4 arms	18/40	A-PRF; A/S-PRF; FDBA + collagen membrane; collagen plug	6 mo	CBCT; histology
Talebi Ardakani et al. (20)	Iran	Double-blind parallel RCT	12/20	Allograft + collagen ± I-PRF	3 mo	CBCT; clinical; histology
Gowda et al. (21)	India	Parallel RCT	16/16	Dentin matrix + A-PRF/i-PRF vs. A-PRF	4 mo	CBCT; clinical
Zampara et al. (22)	Greece	Multiarm RCT	32/32	Allograft; xenograft; alloplast; standard care	NR	Histology; clinical
Mopati et al. (23)	India	Parallel RCT	20/20	Eggshell HA vs. DFDBA, both with PRF	6 mo	Radiographic; clinical
Elfana et al. (24)	Egypt	Parallel RCT	20/20	Whole-tooth graft vs. demineralized dentin graft	4 mo	CBCT; histology
Abad et al. (25)	Spain	Parallel RCT	28/28	L-PRF vs. spontaneous healing	4 mo	CBCT; digital model
Hussain et al. (26)	Iraq	Parallel RCT	32/32	Autogenous dentin biomaterial vs. unassisted healing	NR	Clinical; radiographic; histology
Clark et al. (27)	USA	Parallel RCT, 4 arms	40/40	A-PRF; A-PRF + FDBA; FDBA; blood clot	NR	Histology; micro-CT
Thakkar et al. (28)	India	Parallel RCT	NR/36	DFDBA + PRF vs. DFDBA	NR	Clinical; radiographic
Schnutenhaus et al. (29)	Germany	Parallel RCT	31/NR	Collagen material vs. control	8–11 wk	CBCT; clinical
Ouyyamwongs et al. (30)	Thailand	Split-mouth RCT	12/40	aDTM + PRF vs. PRF	8 wk	Clinical; radiographic
Dhamija et al. (31)	India	Parallel RCT	20/20	DFDBA + PRF vs. DFDBA	6 mo	Clinical; radiographic; histology
Girish Kumar et al. (32)	India	Parallel RCT	48/48	PRF + bone substitute vs. PRF	6 mo	Clinical; radiographic
Ivanova et al. (33)	Bulgaria	RCT, 3 arms	28/NR	A-PRF; A-PRF + allograft; allograft	4 mo	Dimensional; histology
Castro et al. (34)	Belgium	Split-mouth RCT	21/60	L-PRF vs. A-PRF + vs. blood clot	3 mo	CBCT
Demetter et al. (35)	USA	Parallel RCT, 3 arms	59/59	Cortical FDBA; cancellous FDBA; mixed FDBA	18–20 wk	Clinical; histology
Corning and Mealey (36)	USA	Parallel RCT	38/38	FDBA vs. SDBA	12 wk	Clinical; histology
Saito et al. (37)	USA	Multicenter parallel RCT	45/NR	PLGA-coated *β*-TCP vs. comparator graft	16–20 wk	CBCT; histology
Bajaj et al. (38)	India	Split-mouth RCT	20/40	Nano-HA + DBM + T-PRF vs. comparator	16 wk	Clinical; radiographic
Shahdad et al. (39)	UK	Parallel RCT	30/NR	PEG membrane vs. collagen membrane	6 mo	CBCT; clinical
Choi et al. (40)	South Korea	Multicenter RCT	38–39/NR	Moldable bone material vs. particulate bone material	5 mo	Radiographic; histology
Nainoor et al. (41)	India	Split-mouth double-blind RCT	20/40	EnHA + PRF vs. DFDBA + PRF	4 mo	Clinical; radiographic
Barone et al. (42)	Italy	Parallel RCT	40/40	Porcine xenograft + membrane vs. extraction alone	7 mo	Clinical; histology
Nunes et al. (43)	Brazil	Split-mouth RCT	15/30	Synthetic bone substitute + membrane vs. control	6 mo	CBCT; clinical
Azangookhiavi et al. (44)	Iran	Parallel RCT	32/32	FDBA vs. PRF	12 wk	Clinical; radiographic
Schnutenhaus et al. (45)	Germany	Parallel RCT	88/NR	Collagen cone + membrane vs. control	8–11 wk	CBCT; histology; implant stability
Aladmawy et al. (46)	USA	Split-mouth pilot RCT	8/16	Allograft + nonresorbable membrane with vs. without primary closure	6 mo	Histology
Wróbel et al. (47)	Poland	Parallel RCT	10/10	Allograft + membrane vs. xenograft + membrane	6 mo	Micro-CBCT; histology
Barone et al. (48)	Italy/Spain	Multicenter parallel RCT	28/28	Collagenated porcine bone vs. cortical porcine bone	3 mo	Volumetric model analysis

A-PRF, advanced platelet-rich fibrin; CBCT, cone-beam computed tomography; DBM, demineralized bone matrix; DFDBA, demineralized freeze-dried bone allograft; EnHA, eggshell-derived nanohydroxyapatite; FDBA, freeze-dried bone allograft; HA, hydroxyapatite; I-PRF, injectable platelet-rich fibrin; NR, not reported; PRF, platelet-rich fibrin; RCT, randomized controlled trial; SDBA, solvent-dehydrated bone allograft.

### 3.3 Network and component characteristics

The included trials were classified into 17 complete treatment nodes and eight predefined components. The included trials were classified into 17 complete treatment nodes and eight predefined components, as defined in Table 2. The treatment nodes represented natural healing, PRF alone, scaffold-based strategies, scaffold combined with PRF, scaffold combined with barrier membrane, and scaffold combined with PRF and barrier membrane. At the component level, PRF occurred as a standalone component in 8 intervention occurrences and in combination with other components in 21 occurrences; the corresponding counts were 8 and 17 for allograft, 0 and 18 for barrier membrane, 2 and 4 for xenograft, 5 and 9 for alloplast, 3 and 3 for tooth/dentin-derived scaffold, and 3 and 0 for collagen scaffold. Because multi-arm trials could contribute more than one eligible intervention, these counts represent intervention occurrences rather than numbers of unique RCTs (Supplementary Table S5; Figure S4).

**Table 2:** Component definitions for component network meta-analysis.

Component code	Component	Operational definition	Studies, n	Participants, n
REF	Natural healing/blood clot	Reference process without active scaffold, PRF or membrane component	6	234
ALG	Allograft	Freeze-dried, demineralized or solvent-dehydrated allogeneic bone graft	15	456
XG	Xenograft	Bovine or porcine xenogeneic bone substitute	5	140
AP	Alloplast	Synthetic scaffold, including HA, beta-TCP, calcium phosphosilicate or PLGA-coated beta-TCP	5	137
COL	Collagen-based scaffold	Collagen cone, plug or collagen material used as a scaffold	3	137
DT	Tooth/dentin scaffold	Autogenous tooth, dentin matrix or dentin-derived graft	4	80
PRF	PRF adjunct	Any PRF adjunct, including L-PRF, A-PRF/A-PRF+, I-PRF, T-PRF or e-PRF	16	431
MEM	Barrier membrane	Collagen, synthetic, nonresorbable or PRF-derived membrane/barrier	23	640

The treatment network was connected for both primary outcomes. For horizontal ridge-width change, the standard NMA network comprised 16 studies and 12 treatment nodes, whereas the buccal vertical ridge-height network included 14 studies and 10 treatment nodes. Natural healing, PRF-containing strategies and allograft-based interventions constituted the main evidence pathways linking different treatment comparisons (Figures 2, 3; Supplementary Figures S2, S3).

**Figure 2:**
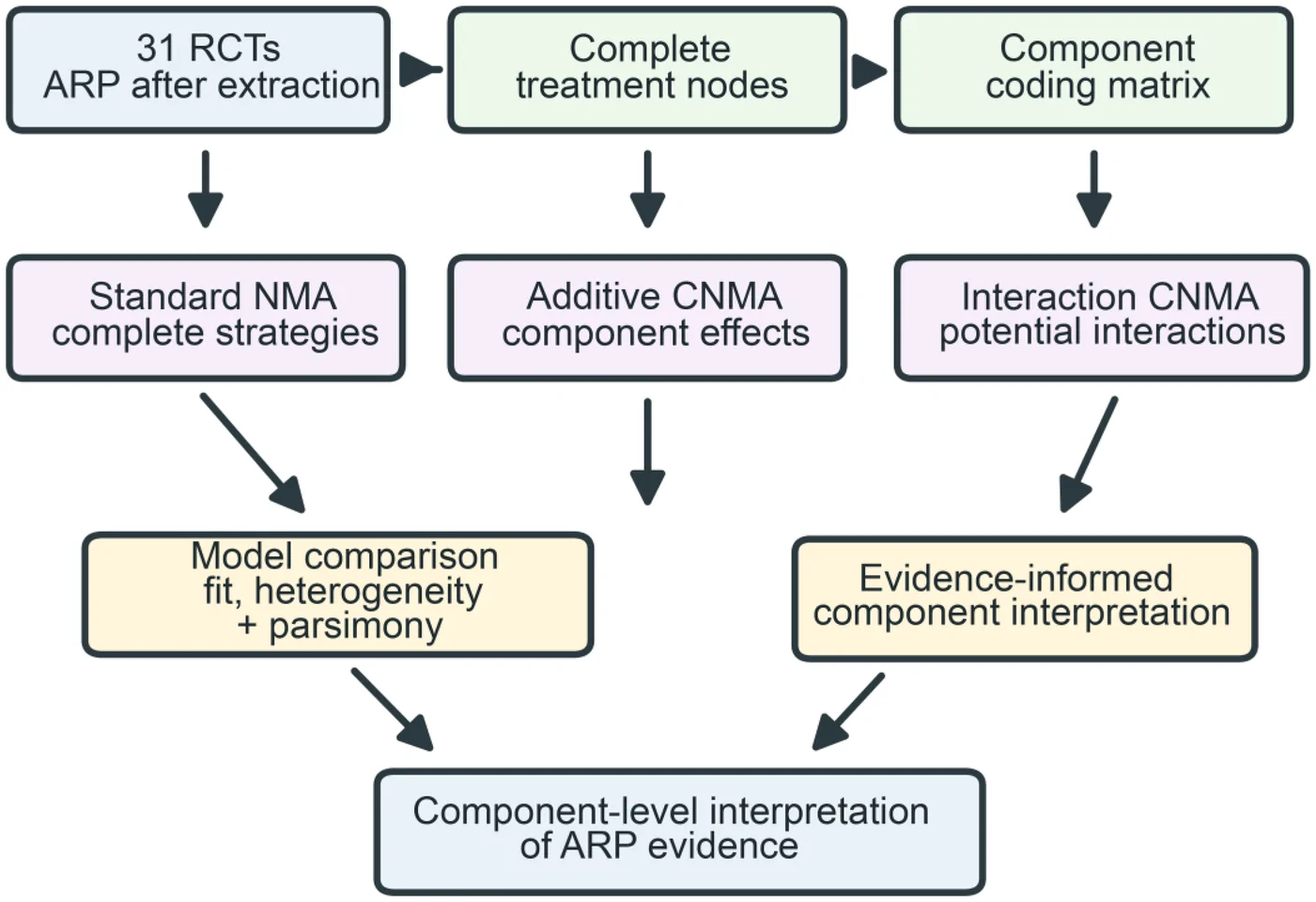
From complete ARP strategies to component-level interpretation.

**Figure 3:**
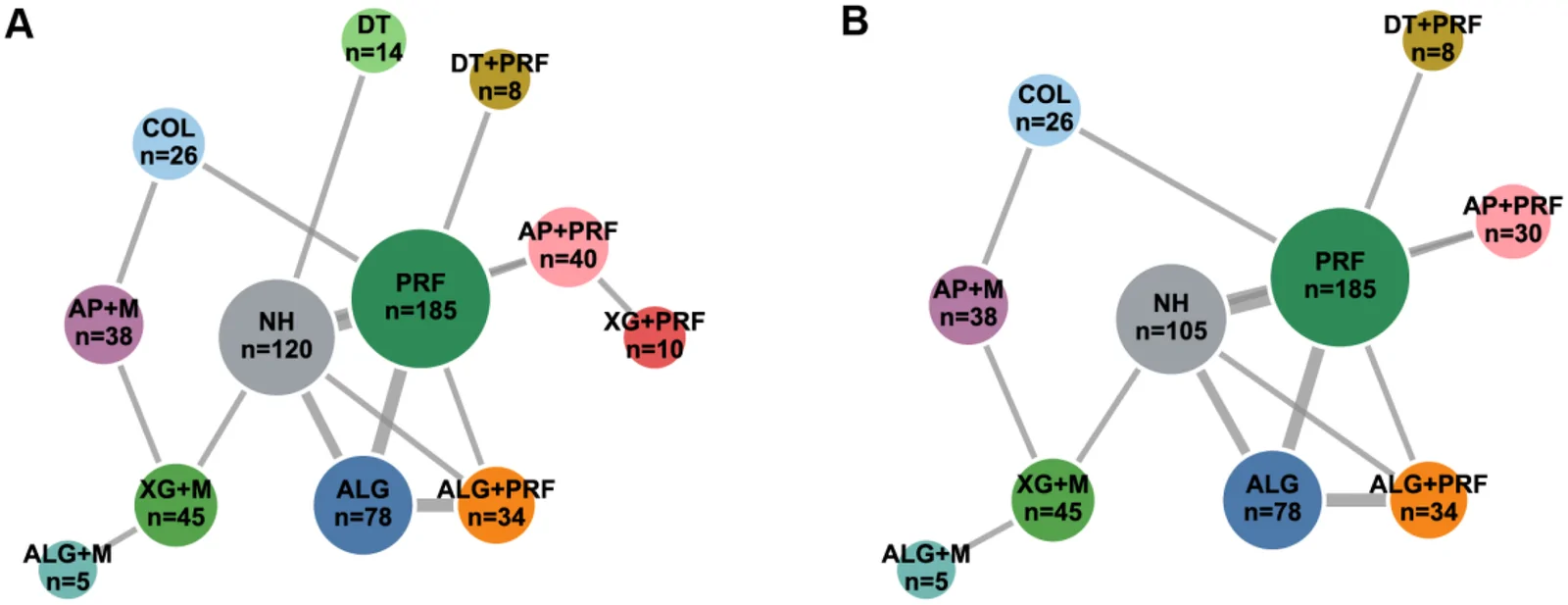
Network geometry for the primary dimensional outcomes.

### 3.4 Risk of bias

Among the 31 included trials, 6 were judged to have a low risk of bias, 20 had some concerns, and 5 were judged to have a high risk of bias. The main sources of uncertainty were related to incomplete reporting of the randomization process and limitations in outcome measurement and blinding. High-risk judgments were predominantly attributed to concerns regarding outcome assessment. For split-mouth trials, additional considerations included the handling of paired data, potential carry-across effects and the independence of extraction sites (Figure 4; Supplementary Table S6; Figure S1).

**Figure 4:**
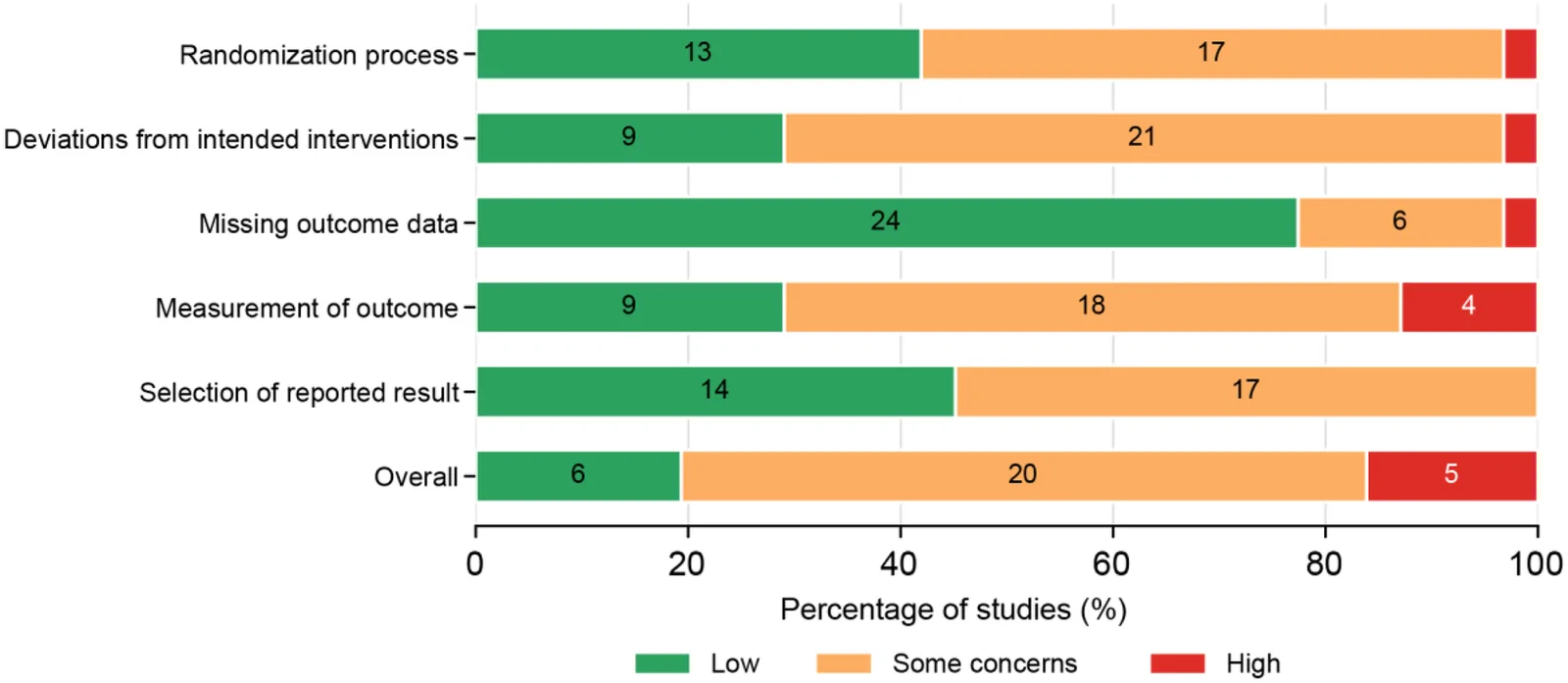
Risk of bias summary for included randomized controlled trials.

### 3.5 Standard network meta-analysis of primary outcomes

For all dimensional outcomes, negative MDs indicated less dimensional loss relative to the comparator and therefore favored ridge preservation.

For horizontal ridge-width change, the random-effects network meta-analysis showed that several complete ARP strategies were associated with reduced ridge dimensional loss compared with natural healing. The largest effects were observed for dentin-derived grafts (MD −2.07 mm, 95% CI −3.17 to −0.97), xenograft combined with barrier membrane (MD −1.78 mm, 95% CI −2.57 to −0.99), and allograft combined with barrier membrane (MD −1.78 mm, 95% CI −3.06 to −0.50). Allograft combined with PRF was also associated with less horizontal ridge-width reduction (MD −1.28 mm, 95% CI −1.93 to −0.63). In contrast, PRF alone showed a favorable direction of effect, although the confidence interval included the null value (Supplementary Tables S7, S8).

For buccal vertical ridge-height change, allograft combined with barrier membrane demonstrated the greatest reduction in vertical ridge-height loss compared with natural healing (MD −3.03 mm, 95% CI −4.86 to −1.20), followed by dentin-derived graft combined with PRF (MD −2.49 mm, 95% CI −3.85 to −1.12) and xenograft combined with barrier membrane (MD −2.23 mm, 95% CI −3.53 to −0.93). Several additional interventions showed directionally favorable effects, although estimates were associated with wider confidence intervals due to limited evidence for some treatment nodes. Complete network estimates and league tables are presented in Figure 5A, Table 3 and Supplementary Tables S9, S10.

**Figure 5:**
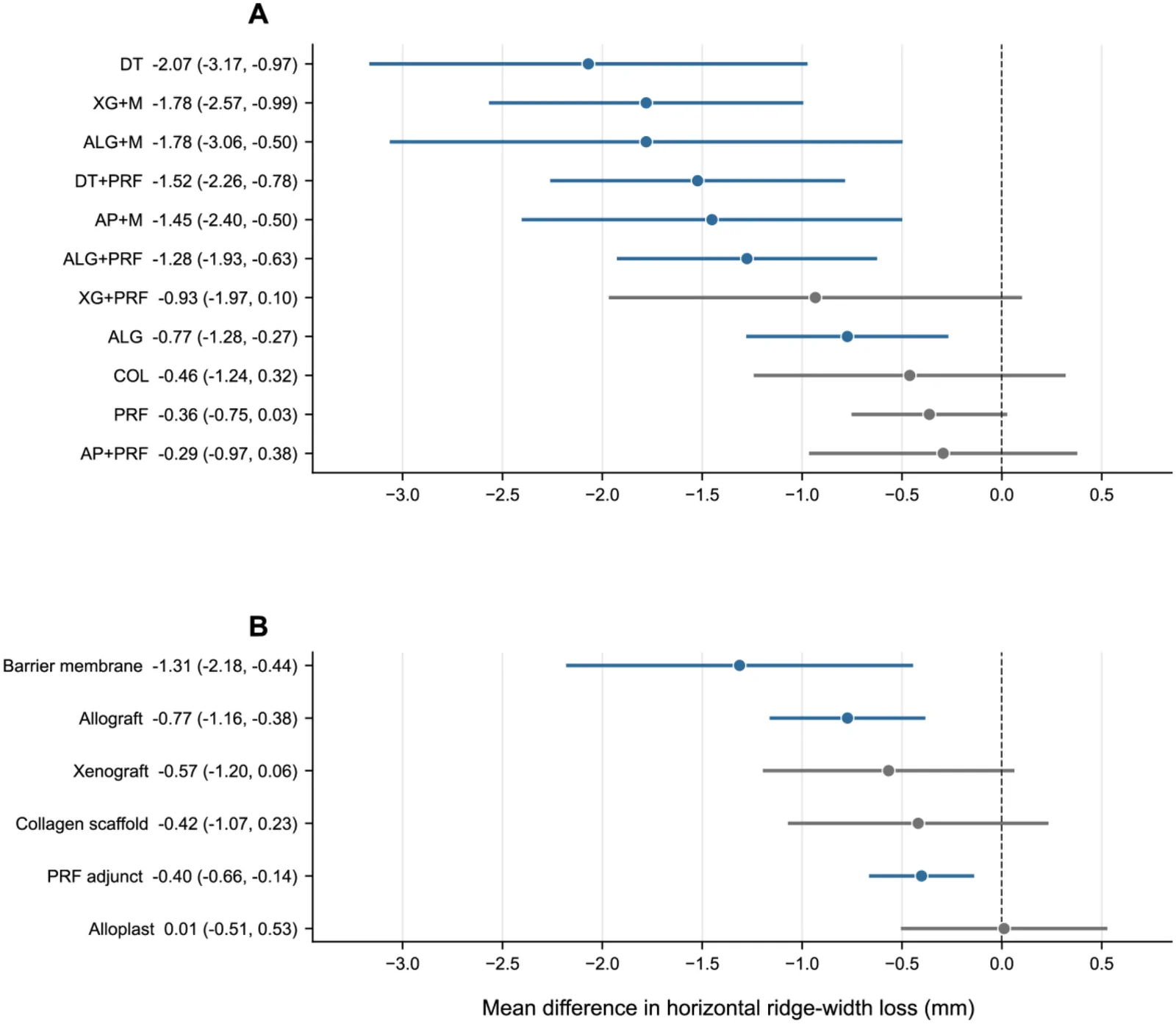
Effects of complete intervention strategies and independent components on horizontal ridge-width preservation.

**Table 3:** Standard network meta-analysis for horizontal ridge-width change .

Rank	Node	Intervention	No. of studies	Sample size	MD vs. natural healing, mm (95% CI)	*P*-score
1	DT	Dentin graft	1	14	−2.07 (−3.17 to −0.97)	0.879
2	XG + M	Xenograft + membrane	3	45	−1.78 (−2.57 to −0.99)	0.818
3	ALG + M	Allograft + membrane	1	5	−1.78 (−3.06 to −0.50)	0.785
4	DT + PRF	Dentin graft + PRF	1	8	−1.52 (−2.26 to −0.78)	0.721
5	AP + M	Alloplast + membrane	2	38	−1.45 (−2.40 to −0.50)	0.678
6	ALG + PRF	Allograft + PRF	3	34	−1.28 (−1.93 to −0.63)	0.620
7	XG + PRF	Xenograft + PRF	1	10	−0.93 (−1.97 to 0.10)	0.469
8	ALG	Allograft	5	78	−0.77 (−1.28 to −0.27)	0.386
9	COL	Collagen scaffold	2	26	−0.46 (−1.24 to 0.32)	0.244
10	PRF	PRF alone or PRF-based adjunct	8	185	−0.36 (−0.75 to 0.03)	0.196
11	AP + PRF	Alloplast + PRF	2	40	−0.29 (−0.97 to 0.38)	0.166
12	NH	Natural healing/blood clot	7	120	Reference	0.036

Effect estimates are mean differences in horizontal ridge-width loss compared with natural healing/blood clot healing. Negative values indicate less ridge-width loss and therefore better ridge preservation. Rankings are based on *P*-scores from the random-effects network meta-analysis.

### 3.6 Component network meta-analysis

#### 3.6.1 Independent component effects

In the additive component network meta-analysis for horizontal ridge-width change, barrier membrane was associated with the largest independent component effect among the evaluated ARP components (MD −1.31 mm, 95% CI −2.18 to −0.44). Allograft also showed a statistically significant component-level association with reduced ridge-width loss (MD −0.77 mm, 95% CI −1.16 to −0.38).

The PRF adjunct component demonstrated a smaller but statistically significant effect (MD −0.40 mm, 95% CI −0.66 to −0.14). Estimates for xenograft (MD −0.57 mm, 95% CI −1.20 to 0.06) and collagen-based scaffold (MD −0.42 mm, 95% CI −1.07 to 0.23) were directionally favorable but did not reach statistical significance. No statistically detectable independent effect was observed for the alloplast component (MD 0.01 mm, 95% CI −0.51 to 0.53). Component-level estimates are presented in Figure 5B, Table 4 and Supplementary Table S11.

**Table 4:** Component effects from additive component network meta-analysis for horizontal ridge-width change.

Component	Incremental MD, mm (95% CI)	*P* value	Preliminary certainty
Xenograft	−0.57 (−1.20 to 0.06)	0.078	Very low
Allograft	−0.77 (−1.16 to −0.38)	<0.001	Low
Alloplast	0.01 (−0.51 to 0.53)	0.966	Very low
Collagen scaffold	−0.42 (−1.07 to 0.23)	0.208	Very low
PRF adjunct	−0.40 (−0.66 to −0.14)	0.003	Low
Barrier membrane	−1.31 (−2.18 to −0.44)	0.003	Low

Component effects were estimated using the additive CNMA model. Negative values indicate reduced horizontal ridge-width loss and therefore greater ridge preservation. Estimates represent the average contribution of each component within the available randomized evidence network and should be interpreted considering uncertainty, heterogeneity and network structure.

#### 3.6.2 Interaction effects

Interaction component network meta-analysis was performed to explore whether the effect of PRF differed according to scaffold type. The estimated interaction effect between xenograft and PRF was 0.28 mm (95% CI −1.21 to 1.76), while the interaction between alloplast and PRF was 0.60 mm (95% CI −0.97 to 2.18). Neither interaction estimate provided statistical evidence of a component-level interaction. The collagen scaffold × PRF interaction could not be estimated because of insufficient connected evidence. Interaction estimates are presented in Figure 6A and Supplementary Table S12.

**Figure 6:**
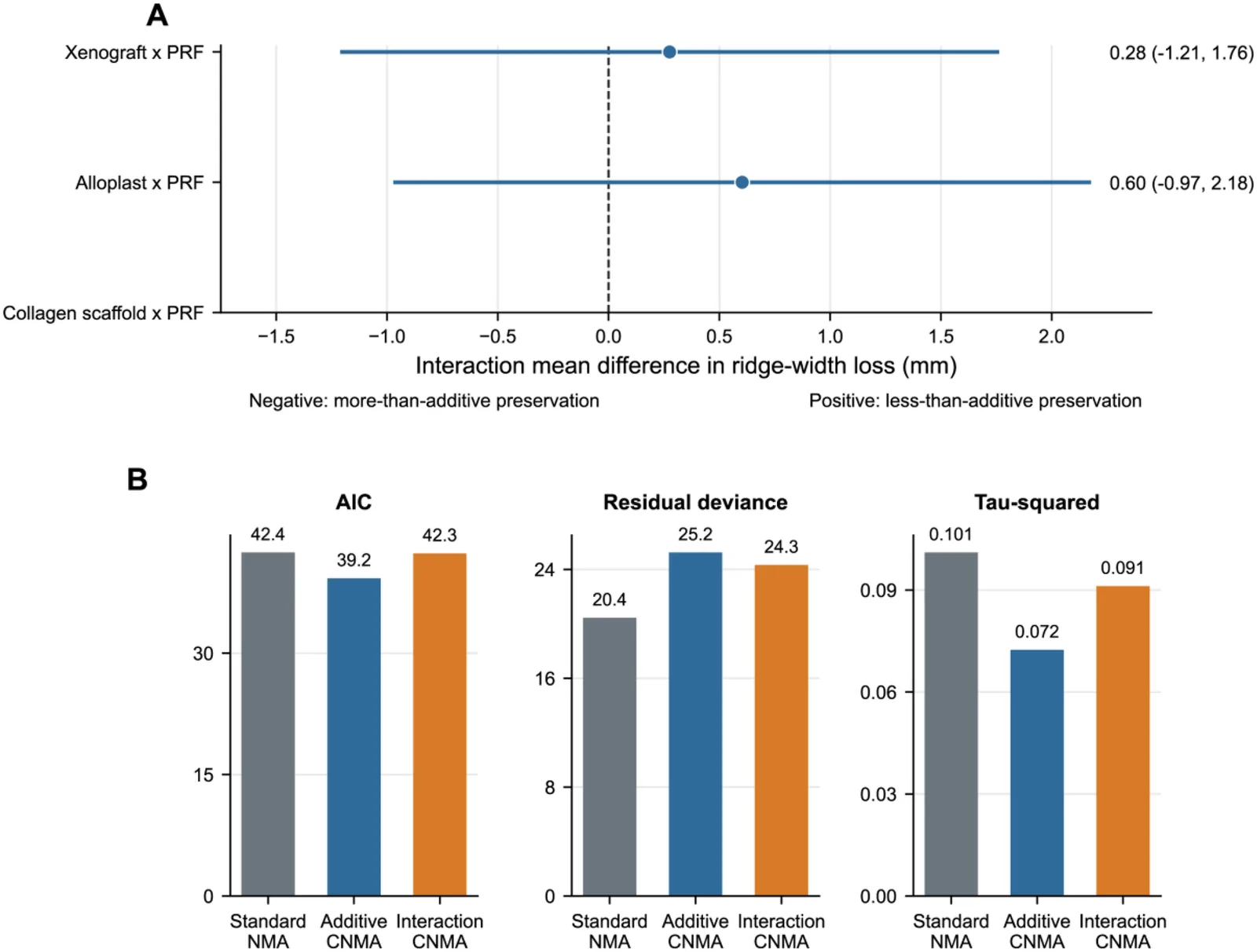
PRF interaction effects and model fit comparison for horizontal ridge-width change. **(A)** Forest plot of prespecified PRF interaction effects from the interaction component network meta-analysis. **(B)** Comparison of standard NMA, additive CNMA, and interaction CNMA according to approximate AIC, residual deviance/Q statistic, and tau-squared. Approximate AIC reflects the balance between model fit and complexity, whereas residual deviance provides a separate measure of absolute model fit; therefore, a lower AIC should not be interpreted as uniformly superior statistical fit. Negative MDs in panel A indicate less ridge-dimensional loss and therefore favor the intervention or component shown.

#### 3.6.3 Model comparison

The additive CNMA showed improved model parsimony compared with the standard NMA and interaction CNMA, with a lower approximate AIC (39.24) and lower between-study variance (*τ*^2^ = 0.072) than the standard NMA (AIC = 42.44; *τ*^2^ = 0.101) and interaction CNMA (AIC = 42.32; *τ*^2^ = 0.091). However, the residual deviance of the additive CNMA was higher than that of the standard NMA, indicating that its lower approximate AIC and reduced between-study variance should not be interpreted as uniformly better absolute model fit. Rather, the lower approximate AIC indicates a more favorable balance between model fit and complexity and supports a more parsimonious representation of the available evidence. The interaction model introduced additional parameters without providing a clear overall advantage across the model-comparison criteria. Model comparison results are summarized in Figure 6B and Supplementary Table S13.

### 3.7 Secondary outcomes

Histomorphometric outcomes were reported in 16 studies, with the most frequently assessed parameters being newly formed bone, residual graft material and connective or non-mineralized tissue. Quantitative synthesis using network or component network meta-analysis was not performed because of substantial variation in outcome definitions, measurement methods, biopsy protocols and follow-up periods. Across the available studies, scaffold-based ARP strategies generally showed greater new bone formation or hard-tissue maturation compared with spontaneous healing; however, the magnitude of these effects varied according to biomaterial characteristics and assessment methods (Supplementary Table S18).

Patient-reported outcomes, including postoperative pain, swelling, early soft-tissue healing and analgesic consumption, were reported inconsistently across trials, with considerable variation in measurement scales and assessment time points. Safety outcomes, including infection, wound dehiscence, membrane exposure and graft loss, were infrequently reported and were generally based on small numbers of events. Owing to the limited and heterogeneous reporting of these outcomes, they were summarized descriptively rather than incorporated into network-level analyses (Supplementary Table S19).

### 3.8 Sensitivity, heterogeneity and inconsistency

Sensitivity analyses showed that the overall pattern of component effects was largely consistent across alternative analytical scenarios. After excluding studies at high risk of bias, barrier membrane and allograft remained associated with favorable component-level estimates, whereas the estimated contribution of PRF remained smaller. Restriction to the prespecified 4–6-month follow-up window resulted in fewer connected comparisons but did not substantially alter the direction of the main findings. Alternative PRF coding strategies, including separate classification of PRF–membrane preparations and other PRF formulations, produced comparable component estimates (Supplementary Table S15).

Network heterogeneity was moderate for the primary outcomes, with variability observed across treatment comparisons. Global inconsistency testing indicated evidence of inconsistency within the primary networks, particularly for horizontal ridge-width change (between-design Q = 19.90, *P* = 0.006). However, node-splitting analyses did not identify statistically significant disagreement between direct and indirect estimates for individual comparisons. Potential contributors to heterogeneity and inconsistency included differences in socket morphology, follow-up duration, outcome measurement methods, scaffold composition, membrane use and PRF preparation protocols (Supplementary Table S14; Figure S5).

Comparison-adjusted funnel plots for the primary dimensional outcomes were examined descriptively for potential small-study effects (Supplementary Figures S6, S7). No formal statistical tests for funnel-plot asymmetry were performed because of the limited number of studies contributing to several treatment nodes and comparisons. Accordingly, the presence of publication bias or small-study effects could not be reliably excluded.

## 4 Discussion

### 4.1 Main findings and clinical interpretation

The main finding of this study is that the effects of alveolar ridge preservation (ARP) are better understood as the combined contributions of individual treatment components rather than the performance of a single preferred intervention protocol. While standard network meta-analysis (NMA) identified several effective scaffolds- and membrane-containing strategies, component network meta-analysis (CNMA) provided additional insight by separating the independent contributions of scaffold materials, barrier membranes and platelet-rich fibrin (PRF).

In the additive CNMA, barrier membrane showed the largest independent contribution to horizontal ridge-width preservation (MD −1.31 mm, 95% CI −2.18 to −0.44), followed by allograft (MD −0.77 mm, 95% CI −1.16 to −0.38). PRF demonstrated a statistically significant but smaller incremental effect (MD −0.40 mm, 95% CI −0.66 to −0.14). These findings suggest that structural components involved in space maintenance and stabilization may contribute more substantially to dimensional preservation than biologic adjuncts alone. However, the results should not be interpreted as evidence that one material is universally superior, because component effects were estimated within an uneven evidence network. Treatment rankings from the standard NMA should be interpreted cautiously because several nodes were supported by only one or a few small trials. *P*-scores reflect the relative ordering of treatments within the available network and should be interpreted alongside the corresponding effect estimates, 95% CIs, the amount of supporting evidence, and evidence certainty, rather than as definitive evidence of treatment superiority.

From a clinical perspective, these findings provide an evidence-informed framework for interpreting the potential contributions of individual components within ARP strategies (3, 49). Within this framework, membrane and scaffold components showed relatively favorable associations with dimensional preservation, whereas PRF appeared to provide a smaller adjunctive contribution (53, 54). The absence of identifiable scaffold–PRF interactions further suggests that the available evidence does not currently support selecting PRF according to a specific scaffold combination.

### 4.2 Comparison with previous evidence and biological interpretation

Previous systematic reviews have demonstrated that ARP reduces post-extraction ridge dimensional changes compared with spontaneous healing (49). Consistent with this evidence, the present NMA showed favorable effects for several graft- and membrane-containing strategies. However, previous pairwise and network meta-analyses mainly compared complete treatment protocols, making it difficult to determine whether observed benefits were driven by graft materials, membranes, biologic adjuncts or their combinations (50–52).

The CNMA framework provides a different interpretation of these findings. A highly ranked treatment protocol may include multiple active components, and treatment-level comparisons cannot determine which component contributes most to the observed effect. In the present analysis, the favorable performance of several complete protocols appeared to be largely associated with membrane and scaffold components rather than PRF alone.

The contribution of barrier membranes is biologically plausible because membranes may stabilize the socket environment, limit soft-tissue collapse and maintain a protected space during early healing (53, 54). Nevertheless, the membrane component represented different barrier materials and clinical approaches, including collagen-based, synthetic and PRF-derived membranes. Therefore, the estimated effect should be interpreted as an average component-level effect rather than evidence supporting a specific membrane type. Similarly, the allograft component showed a consistent favorable association, which may reflect its ability to maintain space and provide a scaffold during early remodeling (50, 51). However, the present findings do not establish superiority of allograft over other graft categories. Differences in evidence volume, component combinations and clinical application may partly explain the precision of the allograft estimate.

PRF has been proposed to enhance angiogenesis, cellular recruitment and early wound healing through its fibrin matrix and bioactive mediators (55). In the present analysis, the estimated PRF component effect of approximately 0.4 mm was statistically detectable but modest in magnitude. Its clinical relevance remains uncertain and may depend on the clinical context; even a small reduction in dimensional loss could be relevant in selected esthetically demanding or anatomically constrained sites. However, the current evidence is insufficient to support PRF as a substitute for scaffold or membrane components when structural support and space maintenance are required. The relatively smaller dimensional effect observed in this analysis does not contradict these biological functions. Rather, it suggests that biologic stimulation alone may have a limited influence on maintaining external ridge dimensions, which are strongly affected by mechanical stability and space preservation. Furthermore, variation among PRF preparation protocols may have contributed to the heterogeneity of the estimated effect (6, 7, 56–58).

### 4.3 Methodological implications of CNMA

Standard NMA allows comparison of multiple treatment strategies but treats each intervention as a complete package (8, 15). In contrast, CNMA decomposes complex interventions into shared components and estimates their individual contributions (9, 10). This distinction is particularly relevant for ARP, where clinical protocols are constructed through combinations of graft materials, membranes and biologic adjuncts. The present analysis demonstrates that treatment rankings from conventional NMA should be interpreted cautiously. A highly ranked protocol may reflect the combined presence of several components rather than the effect of a single intervention. By estimating component-level effects, CNMA provides additional information that may be more directly relevant to clinical decision-making. However, CNMA relies on several assumptions. The additive model assumes that combined interventions can be approximated by the sum of individual component effects. Although the additive model showed better fit and greater parsimony than alternative models, statistical performance does not necessarily indicate biological independence. Component effects may vary according to material properties, surgical technique, timing and unmeasured interactions (9, 10, 15). Similarly, the absence of statistically significant scaffold–PRF interactions should be interpreted as insufficient evidence rather than evidence of no interaction. Future factorial or adequately powered multi-arm trials will be required to determine whether specific scaffold–PRF combinations produce synergistic effects.

### 4.4 Limitations, certainty and future directions

Several limitations should be considered. First, some treatment nodes and components were supported by limited numbers of trials, resulting in imprecise estimates. Second, component categories included heterogeneous materials and techniques, particularly for membranes, scaffolds and PRF preparations. Therefore, the estimated effects represent average effects across broader component classes rather than specific products or protocols. Third, clinical heterogeneity and potential violations of transitivity remain important considerations. Differences in socket morphology, buccal-wall integrity, surgical procedures, measurement methods and follow-up periods may have influenced relative treatment effects. Global inconsistency was detected in the primary networks and may reduce confidence in some treatment- and component-level estimates, even though local node-splitting analyses did not identify statistically significant disagreement for individual comparisons. In addition, several components were supported by sparse or unevenly distributed evidence, and uneven component co-occurrence may limit the ability of the model to separate individual component effects from the treatment combinations in which they were evaluated. Although sensitivity analyses did not materially alter the overall interpretation of the component effects, these limitations reduce confidence in some component-level estimates. Accordingly, the CNMA estimates should be interpreted as evidence-informed average component effects within the observed network rather than as definitive evidence of superiority of individual components. Fourth, several split-mouth trials did not report paired effect estimates or within-participant correlation coefficients; therefore, residual unit-of-analysis issues arising from within-participant dependence cannot be fully excluded. Fifth, publication bias and small-study effects could not be reliably excluded because several treatment nodes and comparisons were supported by relatively few studies, limiting the interpretability of funnel-plot asymmetry. Sixth, the available evidence was insufficient for robust component-level analysis of several clinically relevant outcomes, including histomorphometric parameters, implant-related outcomes, patient-reported outcomes and adverse events. Future ARP trials should adopt standardized outcome definitions, report baseline socket characteristics and describe scaffold, membrane and biologic adjunct components separately. Factorial designs comparing identical scaffolds with and without PRF or membrane use would be particularly valuable for estimating component interactions. Beyond ARP, this study illustrates the potential application of CNMA to other regenerative interventions composed of multiple therapeutic elements. However, meaningful component inference requires transparent reporting, adequate network connectivity and careful evaluation of additivity and transitivity assumptions (9, 10, 15).

## 5 Conclusions

This component network meta-analysis indicates that the effects of alveolar ridge preservation are likely driven by the combined contribution of individual treatment components rather than by any single intervention protocol. This CNMA suggests that barrier membranes and allograft components were associated with relatively favorable component-level estimates for horizontal ridge-width preservation, whereas PRF showed a smaller incremental effect. However, these findings do not constitute definitive evidence of superiority over other regenerative components and should be considered cautiously given network inconsistency, sparse evidence for some components, and the low or very low certainty of several estimates. Available evidence was insufficient to determine whether PRF provides scaffold-specific synergistic effects. These findings support an evidence-informed, component-based interpretation of complex ARP strategies, rather than a definitive clinical treatment hierarchy. These findings support an evidence-informed, component-based interpretation of complex ARP strategies, rather than a definitive clinical treatment hierarchy. (Figure 7). Future randomized trials with standardized outcome reporting and detailed characterization of scaffold, membrane and biologic adjunct components are needed to better define independent and interactive treatment effects.

**Figure 7:**
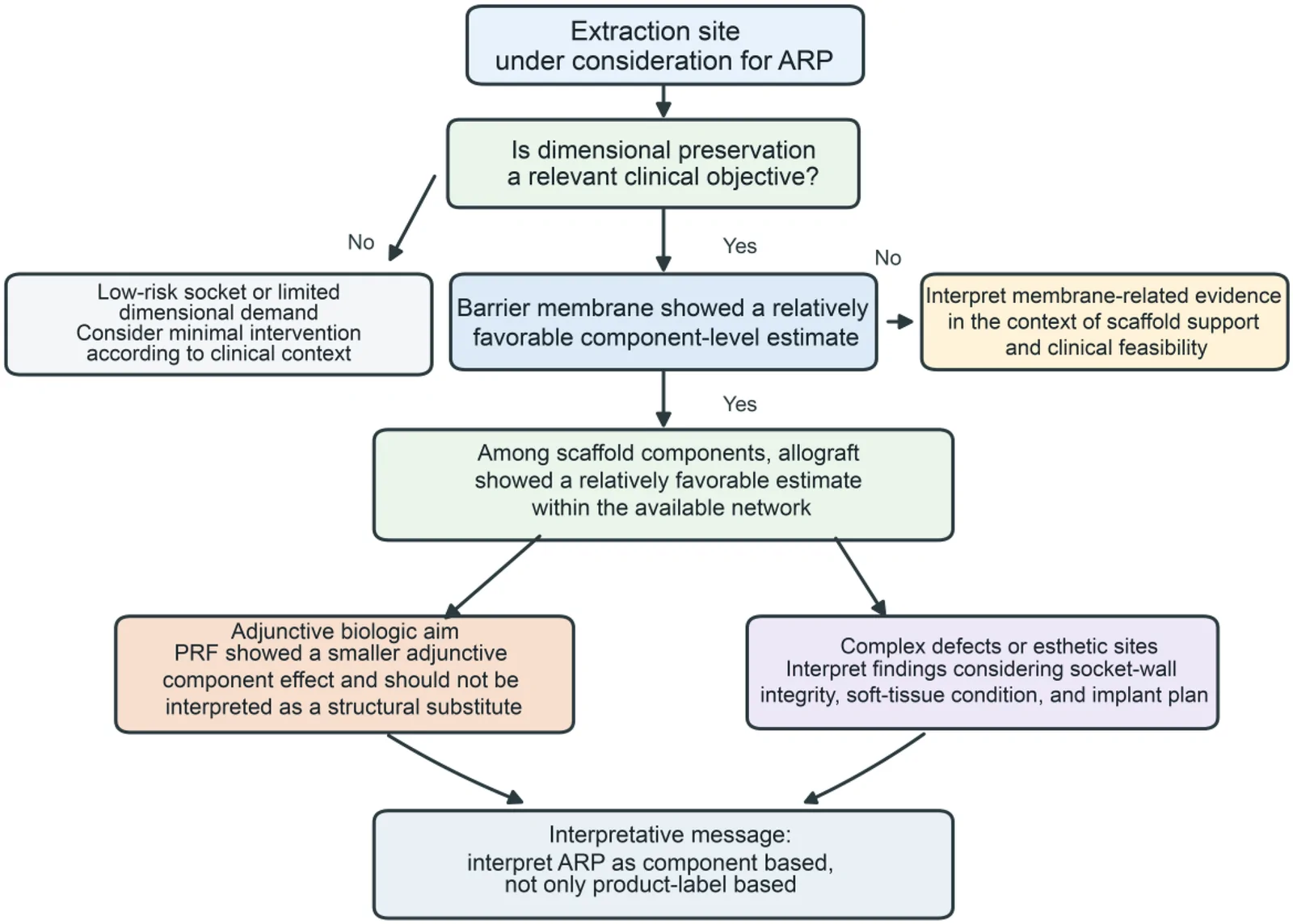
Evidence-informed interpretation of component-level findings for alveolar ridge preservation. Evidence-informed interpretation of component-level findings for alveolar ridge preservation. The framework summarizes the CNMA findings and should not be interpreted as a definitive clinical treatment algorithm or hierarchy.

## Data Availability

The original contributions presented in the study are included in the article/Supplementary Material, further inquiries can be directed to the corresponding author/s.

## References

[1] Araujo MG Lindhe J. Dimensional ridge alterations following tooth extraction. An experimental study in the dog. J Clin Periodontol. (2005) 32(2):212–8. 10.1111/j.1600-051X.2005.00642.x 15691354

[2] Van Der Weijden F Dell'acqua F Slot DE. Alveolar bone dimensional changes of post-extraction sockets in humans: a systematic review. J Clin Periodontol. (2009) 36(12):1048–58. 10.1111/j.1600-051X.2009.01482.x 19929956

[3] Horváth A Mardas N Mezzomo LA Needleman IG Donos N. Alveolar ridge preservation. A systematic review. Clin Oral Investig. (2013) 17(2):341–63. 10.1007/s00784-012-0758-5 22814758

[4] Avila-Ortiz G Elangovan S Kramer KWO Blanchette D Dawson DV. Effect of alveolar ridge preservation after tooth extraction: a systematic review and meta-analysis. J Dent Res. (2014) 93(10):950–8. 10.1177/0022034514541127 24966231 PMC4293706

[5] Iasella Iasella JM Greenwell H Miller RL Hill M Drisko C Bohra AA. Ridge preservation with freeze-dried bone allograft and a collagen membrane compared to extraction alone for implant site development: a clinical and histologic study in humans. J Periodontol. (2003) 74(7):990–9. 10.1902/jop.2003.74.7.990 12931761

[6] Pan J Xu Q Hou J Wu Y Liu Y Li R. Effect of platelet-rich fibrin on alveolar ridge preservation. J Am Dent Assoc. (2019) 150(9):766–78. 10.1016/j.adaj.2019.04.025 31439204

[7] Mir-Mari J Wenz HJ Kim S. Influence of platelet-rich fibrin on alveolar ridge preservation: a systematic review. Int J Oral Maxillofac Surg. (2020) 49(6):769–80. 10.1097/SCS.0b013e31828b6dc3

[8] Lu G Ades AE. Combination of direct and indirect evidence in mixed treatment comparisons. Stat Med. (2004) 23(20):3105–24. 10.1002/sim.1875 15449338

[9] Rücker G Petropoulou M Schwarzer G. Network meta-analysis of multicomponent interventions. Biom J. (2020) 62(3):808–21. 10.1002/bimj.201800167 PMC7217213 31021449

[10] Tsokani S Seitidis G Mavridis D. Component network meta-analysis in a nutshell. BMJ Evid Based Med. (2023) 28(3):183–6. 10.1136/bmjebm-2021-111906 35896417

[11] Page MJ McKenzie JE Bossuyt PM Boutron I Hoffmann TC Mulrow CD. The PRISMA 2020 statement: an updated guideline for reporting systematic reviews. Br Med J. (2021) 372:n71. 10.1136/bmj.n71 PMC8005924 33782057

[12] Hutton B Salanti G Caldwell DM Chaimani A Schmid CH Cameron C. The PRISMA extension statement for reporting of systematic reviews incorporating network meta-analyses of health care interventions: checklist and explanations. Ann Intern Med. (2015) 162(11):777–84. 10.7326/M14-2385 26030634

[13] Sterne JAC Savović J Page MJ Elbers RG Blencowe NS Boutron I. Rob 2: a revised tool for assessing risk of bias in randomised trials. Br Med J. (2019) 366:l4898. 10.1136/bmj.l4898 31462531

[14] Balduzzi S Rücker G Nikolakopoulou A Papakonstantinou T Salanti G Efthimiou O. Netmeta: an R package for network meta-analysis using frequentist methods. J Stat Softw. (2023) 106(2):1–40. 10.18637/jss.v106.i0237138589

[15] Higgins JPT Jackson D Barrett JK Lu G Ades AE White IR. Consistency and inconsistency in network meta-analysis: concepts and models for multi-arm studies. Res Synth Methods. (2012) 3(2):98–110. 10.1002/jrsm.1044 26062084 PMC4433772

[16] Nikolakopoulou A Higgins JPT Papakonstantinou T Chaimani A Del Giovane C Egger M. CINeMA: an approach for assessing confidence in the results of a network meta-analysis. PLoS Med. (2020) 17(4):e1003082. 10.1371/journal.pmed.1003082 32243458 PMC7122720

[17] Brignardello-Petersen R Bonner A Alexander PE Siemieniuk RA Furukawa TA Rochwerg B. Advances in the GRADE approach to rate the certainty in estimates from a network meta-analysis. J Clin Epidemiol. (2018) 93:36–44. 10.1016/j.jclinepi.2017.10.005 29051107

[18] Estrin NE Ahmad P Tran TB Espinoza AR Holmes R Imber J-C. The evolution of extended platelet-rich fibrin membranes for socket grafting: part two: a randomized clinical trial comparing these membranes with collagen membranes. Dent J. (2026) 14(1):45. 10.3390/dj14010045 PMC12840002 41590169

[19] Alrayyes Y Aloraini S Alshagroud R Binrayes A Aljasser R. Extraction and socket preservation before implant placement using freeze-dried allograft (FDBA) and platelet-rich fibrin in smokers: radiographic and histological evaluation. Appl Sci. (2023) 13(12):7076. 10.3390/app13127076

[20] Talebi Ardakani MR Rezaei Esfahrood Z Mashhadiabbas F Hatami M. Comparison of histological, clinical, and radiographic outcomes of postextraction ridge preservation by allogenic bone grafting with and without injectable platelet-rich fibrin: a double-blinded randomized controlled clinical trial. Int J Dent. (2024) 2024(1):8850664. 10.1155/2024/8850664 39483789 PMC11527539

[21] Gowda TM Jayashri M Venkatesh UG Shah R Amanna Kumar BT Deepthi M. Autologous tooth bone graft block compared with advanced platelet-rich fibrin in alveolar ridge preservation: a clinico-radiographic study. J Indian Soc Periodontol. (2023) 27(6):619–25. 10.4103/jisp.jisp_43_23 38434501 PMC10906784

[22] Zampara E Alshammari M De Bortoli J Mullings O Gkisakis IG Benalcázar Jalkh EB. A histologic and histomorphometric evaluation of an allograft, Xenograft, and alloplast graft for alveolar ridge preservation in humans: a randomized controlled clinical trial. J Oral Implantol. (2022) 48(6):541–9. 10.1563/aaid-joi-D-21-00012 35446950

[23] Mopati NRG Ramesh KSV Penmetsa SG Gottumukkala NVSGS Pasupuleti MK Kanakamedala A. A comparative evaluation of eggshell derived hydroxyapatite and demineralized freeze-dried bone allograft in alveolar ridge preservation – a randomized clinical trial. Biomater Investig Dent. (2025) 12:175–83. 10.2340/biid.v12.44941 PMC12706998 41409125

[24] Elfana A El-Kholy S Saleh HA Fawzy El-Sayed K. Alveolar ridge preservation using autogenous whole-tooth versus demineralized dentin grafts: a randomized controlled clinical trial. Clin Oral Implants Res. (2021) 32(5):539–48. 10.1111/clr.13722 33565656

[25] Abad CE Sanz-Sanchez I Serrano V Sanz Esporrin J Sanz-Martin I Sanz M. Efficacy of the application of leukocyte and platelet-rich fibrin (L-PRF) on alveolar ridge preservation. A randomized controlled clinical trial. Clin Implant Dent Relat Res. (2023) 25(3):592–604. 10.1111/cid.13208 37088697

[26] Hussain AA Al-Quisi AF Abdulkareem AA. Efficacy of autogenous dentin biomaterial on alveolar ridge preservation: a randomized controlled clinical trial. BioMed Res Int. (2023) 2023(1):7932432. 10.1155/2023/7932432 38179035 PMC10764647

[27] Clark D Rajendran Y Paydar S Ho S Cox D Ryder M. Advanced platelet-rich fibrin and freeze-dried bone allograft for ridge preservation: a randomized controlled clinical trial. J Periodontol. (2018) 89(4):379–87. 10.1002/JPER.17-0466 29683498 PMC6483085

[28] Thakkar DJ Deshpande NC Dave DH Narayankar SD. A comparative evaluation of extraction socket preservation with demineralized freeze-dried bone allograft alone and along with platelet-rich fibrin: a clinical and radiographic study. Contemp Clin Dent. (2016) 7(3):371. 10.4103/0976-237X.188567 27630503 PMC5004552

[29] Schnutenhaus S Doering I Dreyhaupt J Rudolph H Luthardt RG. Alveolar ridge preservation with a collagen material: a randomized controlled trial. J Periodontal Implant Sci. (2018) 48(4):236. 10.5051/jpis.2018.48.4.236 30202607 PMC6125670

[30] Ouyyamwongs W Leepong N Suttapreyasri S. Alveolar ridge preservation using autologous demineralized tooth matrix and platelet-rich fibrin versus platelet-rich fibrin alone. Implant Dent. (2019) 28(5):455–62. 10.1097/ID.0000000000000918 31188170

[31] Dhamija R Shetty V Vineeth K Nagaraju R Rao RS. Socket preservation with demineralized freeze-dried bone allograft and platelet-rich fibrin for implant site development: a randomized controlled trial. The J Indian Prosthodont Soc. (2020) 20(3):304. 10.4103/jips.jips_2_20 33223701 PMC7654195

[32] Girish Kumar N Chaudhary R Kumar I Arora SS Kumar N Singh H. To assess the efficacy of socket plug technique using platelet rich fibrin with or without the use of bone substitute in alveolar ridge preservation: a prospective randomised controlled study. Oral Maxillofac Surg. (2018) 22(2):135–42. 10.1007/s10006-018-0680-3 29411166

[33] Ivanova V Chenchev I Zlatev S Kanazirski N. Dimensional ridge alterations and histomorphometric analysis following socket preservation with prf or allograft. Randomized controlled clinical study. J IMAB - Annu Proc. (2019) 25(4):2853–61. 10.5272/jimab.2019254.2853

[34] Castro AB Van Dessel J Temmerman A Jacobs R Quirynen M. Effect of different platelet-rich fibrin matrices for ridge preservation in multiple tooth extractions: a split-mouth randomized controlled clinical trial. J Clin Periodontol. (2021) 48(7):984–95. 10.1111/jcpe.13463 33847018

[35] Demetter RS Calahan BG Mealey BL. Histologic evaluation of wound healing after ridge preservation with cortical, cancellous, and combined cortico-cancellous freeze-dried bone allograft: a randomized controlled clinical trial. J Periodontol. (2017) 88(9):860–8. 10.1902/jop.2017.170155 28452622

[36] Corning PJ Mealey BL. Ridge preservation following tooth extraction using mineralized freeze-dried bone allograft compared to mineralized solvent-dehydrated bone allograft: a randomized controlled clinical trial. J Periodontol. (2019) 90(2):126–33. 10.1002/JPER.18-0199 30161278

[37] Saito H Couso-Queiruga E Shiau HJ Stuhr S Prasad H Allareddy TV. Evaluation of poly lactic-co-glycolic acid-coated *β*-tricalcium phosphate for alveolar ridge preservation: a multicenter randomized controlled trial. J Periodontol. (2021) 92(4):524–35. 10.1002/JPER.20-0360 32996128

[38] Bajaj P Dhadse PV Patil RT Salian SS Punse SD Oza R. Evaluation of effectiveness of nanocrystalline hydroxyapatite and demineralized bone matrix combined with Titanium-platelet rich fibrin for ridge preservation: a randomized controlled clinical trial. J Contemp Dent Pract. (2025) 25(11):1069–76. 10.5005/jp-journals-10024-3786 39905614

[39] Shahdad S Gamble E Matani J Zhang L Gambôa A. Randomized clinical trial comparing PEG-based synthetic to porcine-derived collagen membrane in the preservation of alveolar bone following tooth extraction in anterior maxilla. Clin Oral Implants Res. (2020) 31(10):1010–24. 10.1111/clr.13648 32799365

[40] Choi Y Cheong J Song Y Kim J Kang D-Y Ahn H-S. Moldable and particulate bone material in alveolar ridge preservation: a multicenter randomized controlled trial. Clin Implant Dent Relat Res. (2025) 27(2):e70011. 10.1111/cid.70011 40070028 PMC11951245

[41] Nainoor N Shobha ES Prashanth NT Rangan V Malick R Shaju N. Comparison of clinical and radiographic assessment of efficacy of eggshell-derived nanohydroxyapatite (EnHA) and demineralized freeze-dried bone allograft (DFDBA), both covered with platelet-rich fibrin (PRF) as a barrier membrane in socket preservation: a randomized controlled clinical trial. J Maxillofac Oral Surg. (2026) 25(1):225–37. 10.1007/s12663-024-02187-4 41648709 PMC12868432

[42] Barone A Aldini NN Fini M Giardino R Calvo Guirado JL Covani U. Xenograft versus extraction alone for ridge preservation after tooth removal: a clinical and histomorphometric study. J Periodontol. (2008) 79(8):1370–7. 10.1902/jop.2008.070628 18672985

[43] Nunes FAS Pignaton TB Novaes AB Taba M Messora MR Palioto DB. Evaluation of a bone substitute covered with a collagen membrane for ridge preservation after tooth extraction. Clinical and tomographic randomized controlled study in humans. Clin Oral Implants Res. (2018) 29(4):424–33. 10.1111/clr.13140 29569754

[44] Azangookhiavi H Ghodsi S Jalil F Dadpour Y. Comparison of the efficacy of platelet-rich fibrin and bone allograft for alveolar ridge preservation after tooth extraction: a clinical trial. Frontiers in Dentistry. (2020) 17(1):1–6. 10.18502/fid.v17i1.3961 33615286 PMC7882193

[45] Schnutenhaus S Götz W Dreyhaupt J Rudolph H Luthardt RG Edelmann C. Associations among primary stability, histomorphometric findings, and bone density: a prospective randomized study after alveolar ridge preservation with a collagen cone. Dent J. (2020) 8(4):112. 10.3390/dj8040112 PMC7712570 33023224

[46] Aladmawy MA Natto ZS Kreitzer M Ogata Y Hur Y. Histological and histomorphometric evaluation of alveolar ridge preservation using an allograft and nonresorbable membrane with and without primary closure: a pilot randomized controlled clinical trial. Medicine (Baltimore). (2022) 101(26):e29769. 10.1097/MD.0000000000029769 35777057 PMC9239639

[47] Wróbel P Jędzierowska M Piecuch A Bąk M Adamczyk J Pławecki P. Evaluation of the effectiveness of the socket preservation technique using allogeneic and Xenogeneic materials: a randomized controlled trial. J Funct Biomater. (2026) 17(6):262. 10.3390/jfb17060262 PMC13300802 42346653

[48] Barone A Toti P Quaranta A Alfonsi F Cucchi A Calvo-Guirado JL. Volumetric analysis of remodelling pattern after ridge preservation comparing use of two types of xenografts. A multicentre randomized clinical trial. Clin Oral Implants Res. (2016) 27(11):e105–15. 10.1111/clr.12572 25727443

[49] Avila-Ortiz G Chambrone L Vignoletti F. Effect of alveolar ridge preservation interventions following tooth extraction: a systematic review and meta-analysis. J Clin Periodontol. (2019) 46(Suppl 21):195–223. 10.1111/jcpe.13057 30623987

[50] Iocca O Farcomeni A Pardiñas Lopez S Talib HS. Alveolar ridge preservation after tooth extraction: a Bayesian network meta-analysis of grafting materials efficacy on prevention of bone height and width reduction. J Clin Periodontol. (2017) 44(1):104–14. 10.1111/jcpe.12633 27712001

[51] Canellas JVS Soares BN Ritto FG Vettore MV Vidigal Júnior GM Fischer RG. What grafting materials produce greater alveolar ridge preservation after tooth extraction? A systematic review and network meta-analysis. J Craniomaxillof Surg. (2021) 49(11):1064–71. 10.1016/j.jcms.2021.06.005 34176715

[52] Martins JR Wagner TP Vallim AC Konflanz W Schwendicke F Celeste RK. Comparison of the efficacy of different techniques to seal the alveolus during alveolar ridge preservation: meta-regression and network meta-analysis. J Clin Periodontol. (2022) 49(7):694–705. 10.1111/jcpe.13628 35451071

[53] Hämmerle CHF Jung RE. Bone augmentation by means of barrier membranes. Periodontol 2000. (2003) 33:36–53. 10.1046/j.0906-6713.2003.03304.x 12950840

[54] Dahlin C Linde A Gottlow J Nyman S. Healing of bone defects by guided tissue regeneration. Plast Reconstr Surg. (1988) 81(5):672–6. 10.1097/00006534-198805000-00004 3362985

[55] Dohan Ehrenfest DM Rasmusson L Albrektsson T. Classification of platelet concentrates: from pure platelet-rich plasma to leucocyte- and platelet-rich fibrin. Trends Biotechnol. (2009) 27(3):158–67. 10.1016/j.tibtech.2008.11.009 19187989

[56] Siawasch SAM Yu J Castro AB Dhondt R Teughels W Temmerman A. Autologous platelet concentrates in alveolar ridge preservation: a systematic review with meta-analyses. Periodontol 2000. (2025) 97(1):104–30. 10.1111/prd.12609 39345008 PMC11808431

[57] Arora M McAulay N Farag A Natto ZS Lu J Albuquerque R. The effectiveness of platelet rich fibrin in alveolar ridge reconstructive or guided bone regenerative procedures: a systematic review and meta-analysis. J Dent. (2025) 153:105548. 10.1016/j.jdent.2024.105548 39736391

[58] Yan J Lu K. The efficacy of platelet-rich fibrin in alveolar ridge preservation: a systematic review and meta-analysis of randomized controlled trials. Front Med (Lausanne). (2026) 13:1753189. 10.3389/fmed.2026.1753189 41797764 PMC12960125

