# Supplementary material for: Component network meta-analysis of scaffold materials, platelet-rich fibrin and barrier membranes for alveolar ridge preservation
Source: Front Oral Health. 2026 Sep 18;7:1949538. doi: 10.3389/froh.2026.1949538 (PMC13630656; doi:10.3389/froh.2026.1949538)
Supplement: Supplementary file 1 [file Datasheet1.pdf]

Supplementary Material

Component network meta-analysis of scaffold materials, platelet-rich fibrin and barrier membranes for alveolar ridge preservation

Jian Zhang[1], Jie Zhong[1]†, Shihan Huang[1], Ling Zhang[1]*

[1] The Yuexin Dental Clinic, Shengzhou, Shaoxing 312400, Zhejiang Province, China

* Correspondence to:

Ling Zhang, PhD, Doctor

The Yuexin Dental Clinic

Shengzhou, Shaoxing, Zhejiang Province, China

E-mail: 15958590464@163.com

Supplementary Figures

Study	D1	D2	D3	D4	D5	Overall
Estrin et al. 2026	SC	SC	L	SC	SC	SC
Alrayyes et al. 2023	SC	SC	L	SC	SC	SC
Talebi Ardakani et al. 2024	L	L	L	SC	L	SC
Gowda et al. 2023	SC	SC	L	SC	SC	SC
Zampara et al. 2022	SC	SC	L	SC	SC	SC
Gokul et al. 2025	SC	SC	SC	SC	SC	SC
Elfana et al. 2021	SC	SC	L	SC	SC	SC
Abad et al. 2023	SC	SC	L	SC	L	SC
Hussain et al. 2023	SC	SC	SC	SC	SC	SC
Clark et al. 2018	L	SC	SC	L	L	SC
Thakkar et al. 2016	SC	SC	L	SC	SC	SC
Schnutenhaus et al. 2018	L	L	L	L	L	L
Ouyyamwongs et al. 2019	SC	SC	L	H	SC	H
Dhamija et al. 2020	SC	SC	L	SC	SC	SC
Girish Kumar et al. 2018	SC	SC	L	SC	SC	SC
Ivanova et al. 2019	SC	SC	SC	SC	SC	SC
Castro et al. 2021	L	SC	L	SC	L	SC
Demetter et al. 2017	L	SC	SC	L	L	SC
Corning and Mealey 2019	L	L	SC	L	L	SC
Saito et al. 2021	L	L	L	L	L	L
Patil et al. 2024	SC	SC	L	H	SC	H
Shahdad et al. 2020	SC	SC	L	SC	SC	SC
Choi et al. 2025	L	SC	L	SC	L	SC
Nainoor et al. 2026	L	L	L	SC	L	SC
Barone et al. 2008	L	L	L	L	L	L
Nunes et al. 2018	SC	SC	L	H	SC	H
Azangookhiavi et al. 2020	L	L	L	L	L	L
Schnutenhaus et al. 2020	L	L	L	L	L	L
Aladmawy et al. 2022	H	SC	L	H	SC	H
Wrobel et al. 2026	SC	H	H	SC	SC	H
Barone et al. 2016	L	L	L	L	L	L

L = Low risk SC = Some concerns H = High risk

Figure S1. Study-level risk-of-bias traffic-light plot

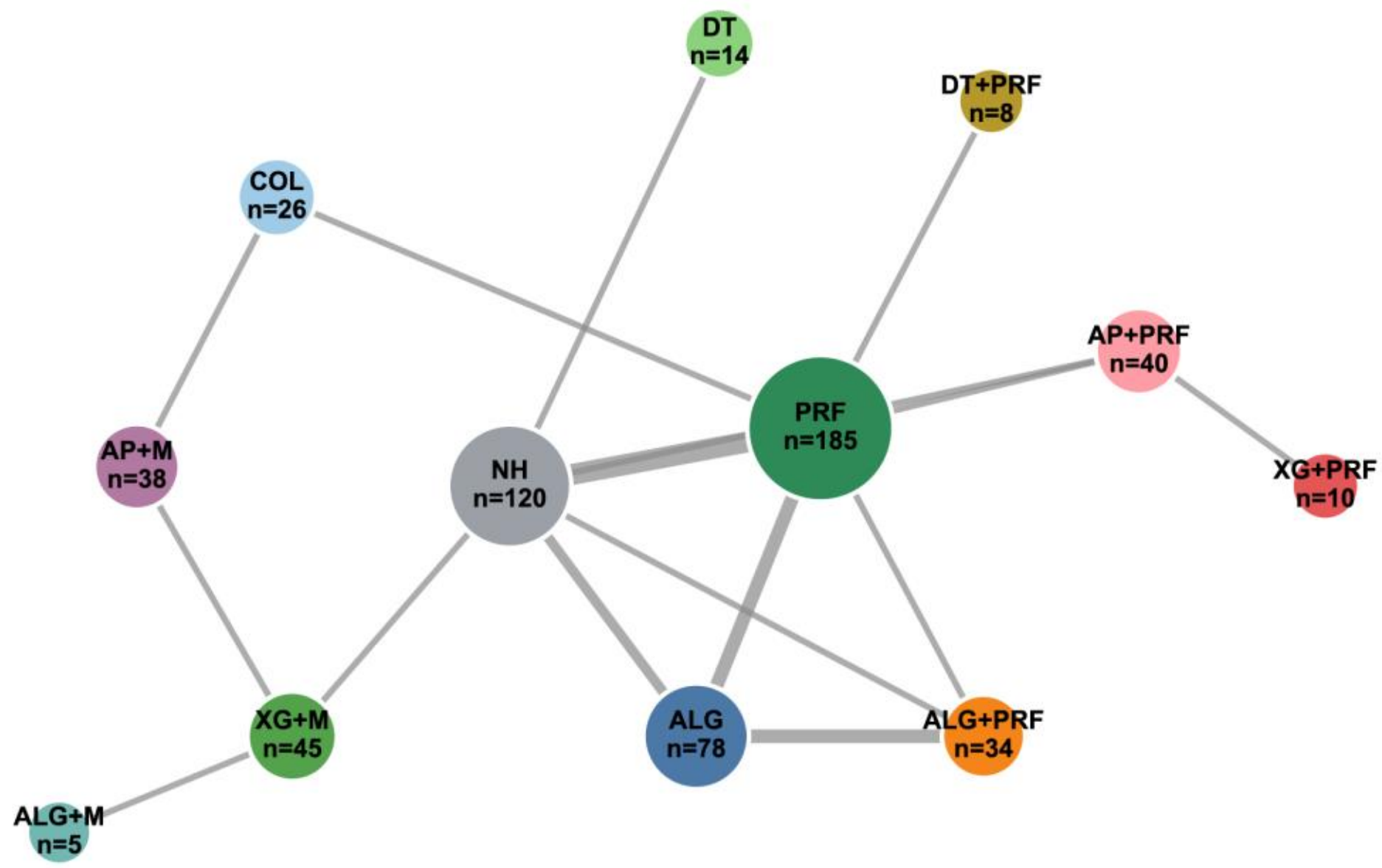

Figure S2. Detailed network geometry for horizontal ridge-width change

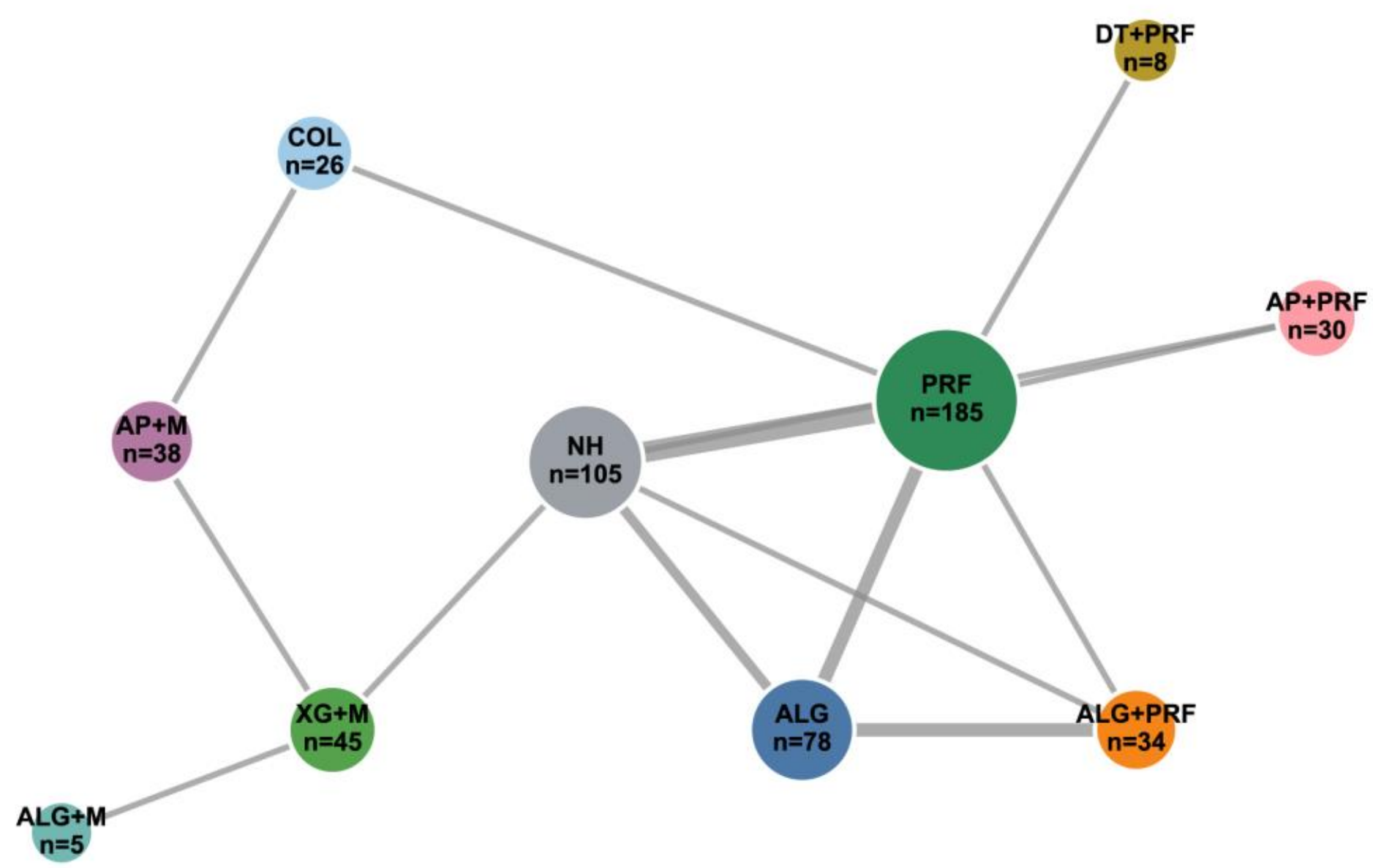

Figure S3. Detailed network geometry for buccal vertical ridge-height change

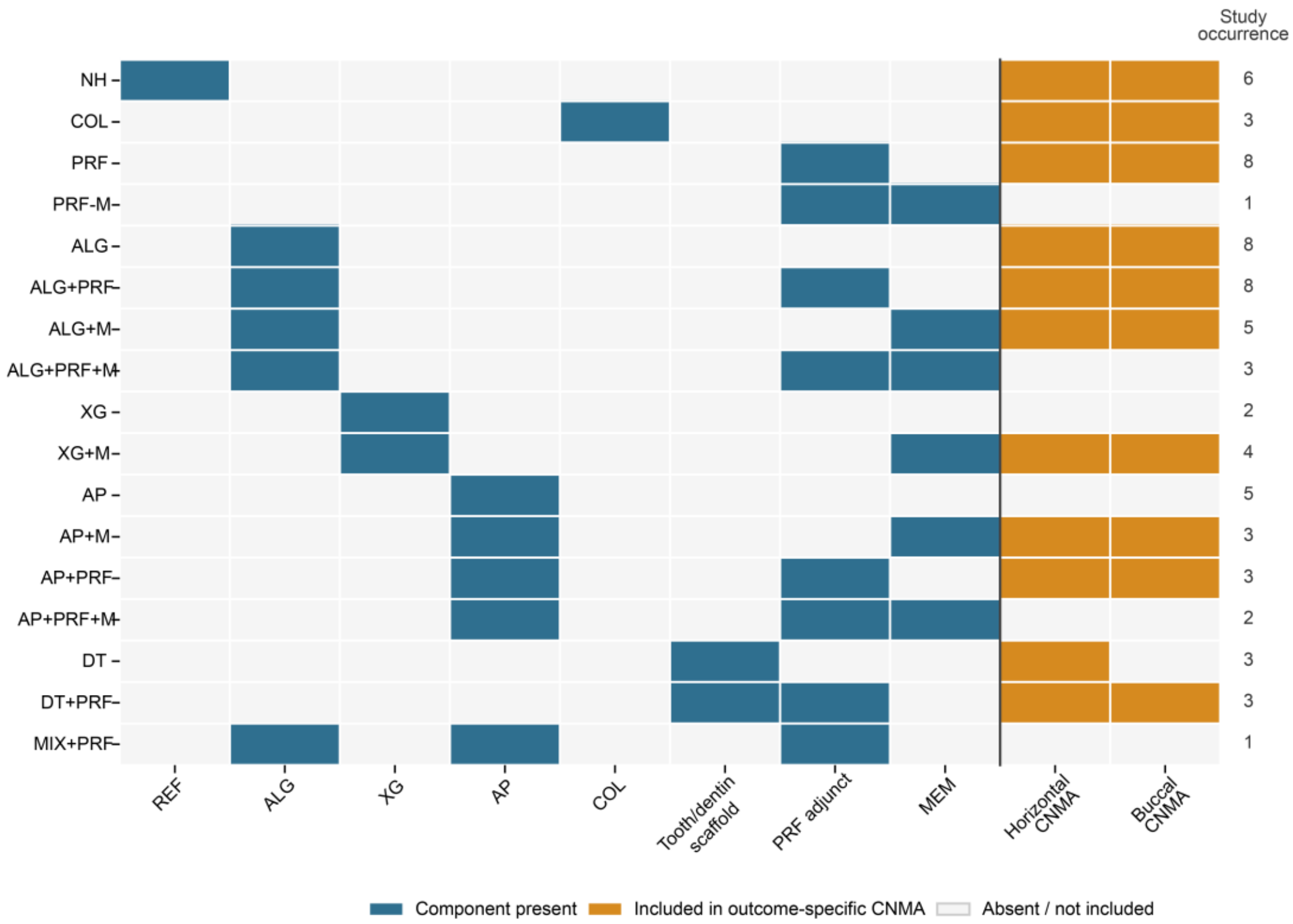

Figure S4. Component-coding matrix for complete ARP treatment nodes. Blue cells indicate the presence of each treatment component, and orange cells indicate whether the treatment node contributed to the horizontal or buccal vertical primary CNMA network. Study occurrence represents intervention occurrences rather than unique RCTs because multi-arm studies could contribute more than one eligible intervention.

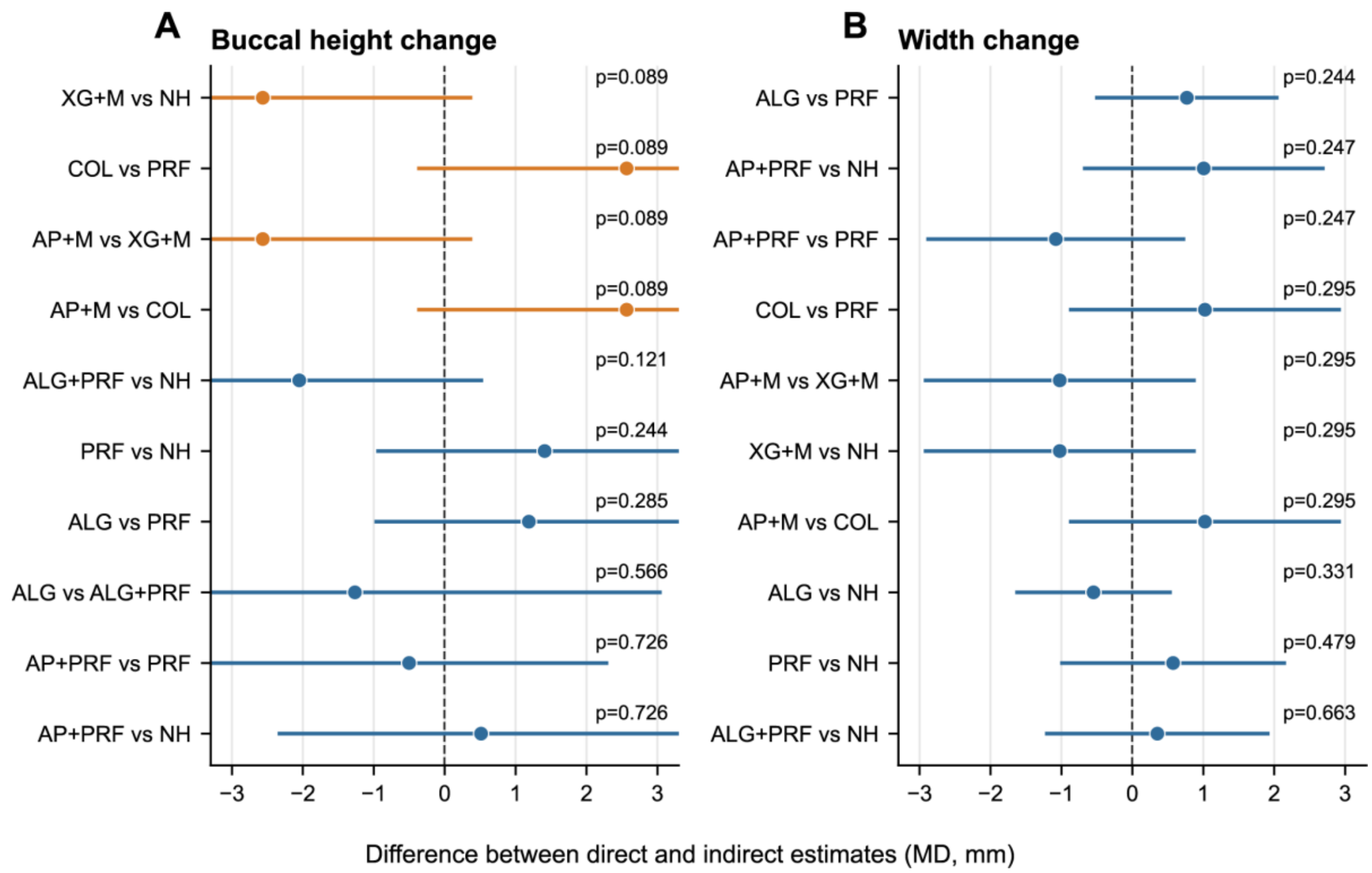

Figure S5. Node-splitting inconsistency plot

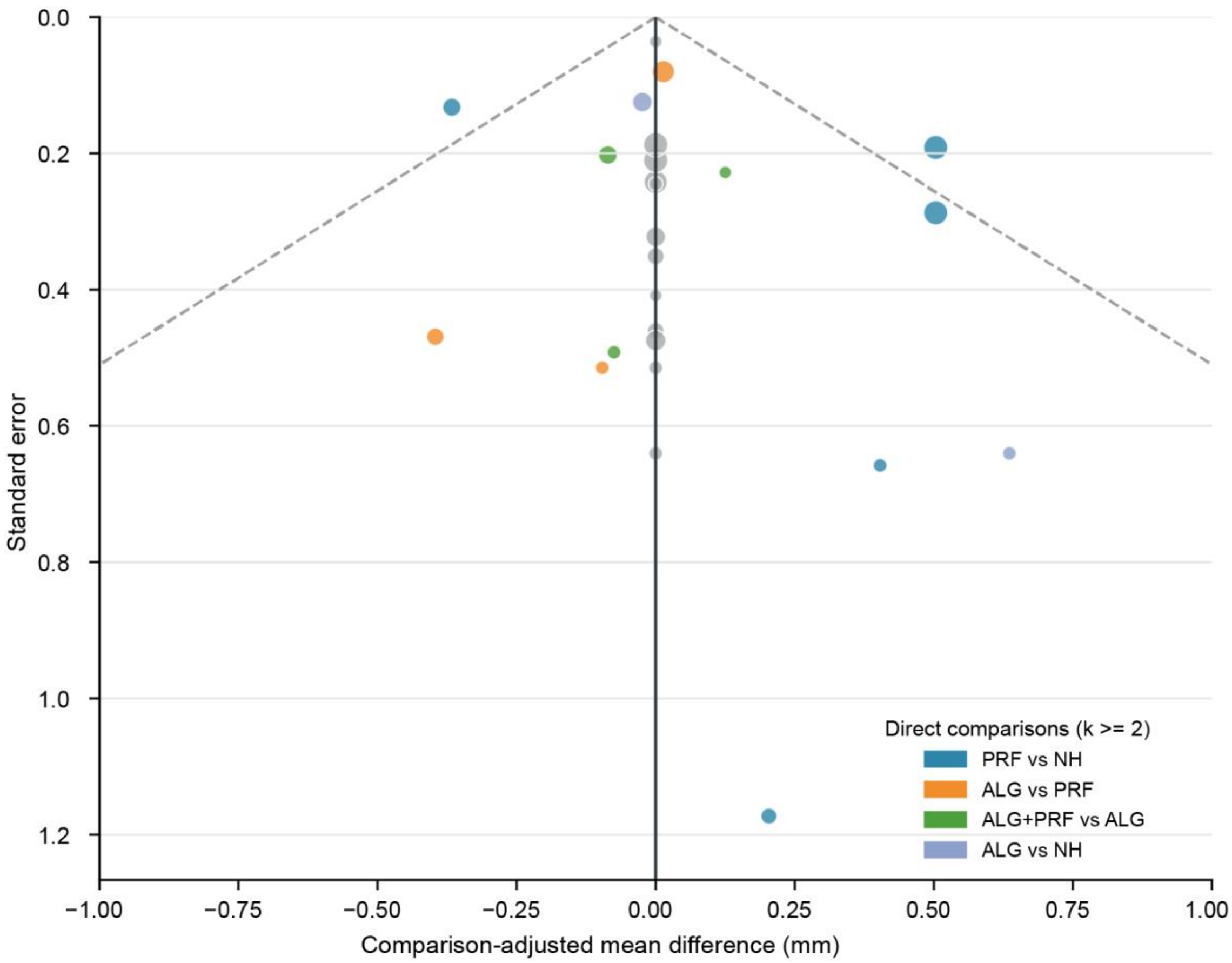

Figure S6. Comparison-adjusted funnel plot for horizontal ridge-width change

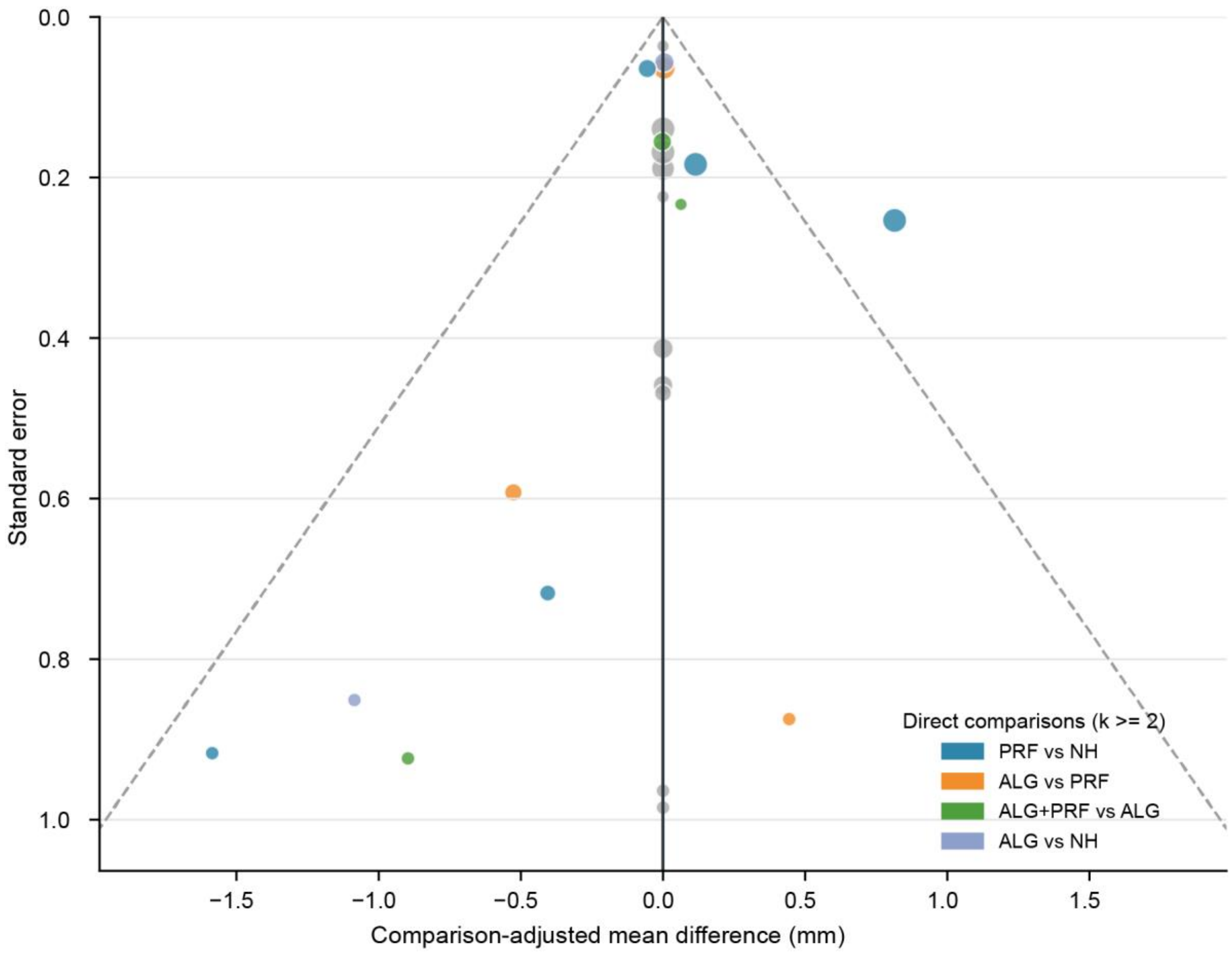

Figure S7. Comparison-adjusted funnel plot for buccal vertical ridge-height change

Supplementary Table

Table S1. Full electronic search strategies and search results for all databases

Database	Search strategy
PubMed	(("alveolar ridge preservation"[Title/Abstract] OR "socket preservation"[Title/Abstract] OR "extraction socket"[Title/Abstract] OR "ridge preservation"[Title/Abstract] OR "socket grafting"[Title/Abstract]) AND ("platelet-rich fibrin"[Title/Abstract] OR PRF[Title/Abstract] OR L-PRF[Title/Abstract] OR A-PRF[Title/Abstract] OR i-PRF[Title/Abstract] OR T-PRF[Title/Abstract] OR CGF[Title/Abstract] OR PRGF[Title/Abstract] OR "bone graft"[Title/Abstract] OR xenograft[Title/Abstract] OR allograft[Title/Abstract] OR alloplast[Title/Abstract] OR "dentin graft"[Title/Abstract] OR collagen[Title/Abstract] OR membrane[Title/Abstract]) AND (randomized[Title/Abstract] OR randomised[Title/Abstract] OR "randomized controlled trial"[Publication Type] OR trial[Title/Abstract]))
Cochrane CENTRAL	("alveolar ridge preservation" OR "socket preservation" OR "extraction socket" OR "ridge preservation" OR "socket grafting") AND ("platelet-rich fibrin" OR PRF OR L-PRF OR A-PRF OR i-PRF OR T-PRF OR CGF OR PRGF OR "bone graft" OR xenograft OR allograft OR alloplast OR "dentin graft" OR collagen OR membrane)
Embase	('alveolar ridge preservation':ti,ab OR 'socket preservation':ti,ab OR 'extraction socket':ti,ab OR 'ridge preservation':ti,ab OR 'socket grafting':ti,ab) AND ('platelet rich fibrin':ti,ab OR prf:ti,ab OR 'l-prf':ti,ab OR 'a-prf':ti,ab OR 'i-prf':ti,ab OR 't-prf':ti,ab OR cgf:ti,ab OR prgf:ti,ab OR 'bone graft':ti,ab OR xenograft:ti,ab OR allograft:ti,ab OR alloplast:ti,ab OR 'dentin graft':ti,ab OR collagen:ti,ab OR membrane:ti,ab) AND (random*:ti,ab OR trial:ti,ab)
Web of Science	TS=("alveolar ridge preservation" OR "socket preservation" OR "extraction socket" OR "ridge preservation" OR "socket grafting") AND TS=("platelet-rich fibrin" OR PRF OR L-PRF OR A-PRF OR i-PRF OR T-PRF OR CGF OR PRGF OR "bone graft" OR xenograft OR allograft OR alloplast OR "dentin graft" OR collagen OR membrane) AND TS=(random* OR trial)

Table S2. Articles excluded during final eligibility refinement after preliminary ARP-RCT screening

Study	Title	Journal/source	Reason for exclusion
Clark et al., 2016 [1]	Platelet-rich fibrin for ridge preservation: a randomized controlled clinical study	Journal of dental research	Excluded from subsequent analysis according to full-text screening decision: Platelet-rich fibrin for ridge preservation: a randomized controlled clinical study.
Rolotti et al., 2025 [2]	A Pilot Study Comparing Dimensional Alterations After Alveolar Ridge Preservation with Xenograft Versus Allograft.	The International journal of periodontics & restorative dentistry	Excluded from subsequent analysis according to full-text screening decision: A Pilot Study Comparing Dimensional Alterations After Alveolar Ridge Preservation with Xenograft Versus Allograft.
Ibrahim et al., 2022 [3]	Comparative Evaluation of Bone Dimensional Changes in Extraction Sockets Preserved With Calcium Phosphosilicate Bone Substitutes With and Without Platelet-Rich Fibrin: A Randomized Controlled Clinical Trial.	The International journal of periodontics & restorative dentistry	Excluded from subsequent analysis according to full-text screening decision: Comparative Evaluation of Bone Dimensional Changes in Extraction Sockets Preserved With Calcium Phosphosilicate Bone Substitutes With and Without Platelet-Rich Fibrin.

Table S3. Clinical and socket-site characteristics relevant to transitivity

Author	Study design	Sample size	No. of tooth	Age	Smoking	Maxilla/mand	Anterior/poster	Socket integrity	Bucca	Flap	Prima	Unit of	Analysis	Follow-up

			sites/sockets		status	ible	ior		l-wall thickness	elevation	ry closure	randomization	unit	included in meta-analysis
Estrin et al. [4]	Single-center, parallel-group RCT; five arms	55	55 extraction sockets	67.3 - 10.5 years	Excluded active smokers	NR	Posterior	NR	NR	NR	NR	participant/site as reported	extraction site/socket or participant as reported	3 months; wound healing at 2 weeks
Alrayyes et al. [5]	Randomized clinical trial; four arms	18	40 upper molar extraction sites	Mean 38 years	All current heavy smokers	Maxilla	Posterior	NR	NR	NR	NR	participant/site as reported	extraction site/socket or participant as reported	6 months
Talebi Ardakani et al. [6]	Parallel, double-blinded RCT; two arms	12	20 extraction sockets	42 - 18 years	NR	Maxilla and mandible	NR	NR	NR	NR	NR	participant/site as reported	extraction site/socket or participant as reported	3 months
Gowda et al. [7]	Parallel RCT; two arms	16	16 molar extraction sites	20-55 years	NR	NR	Posterior	Defective/dehiscence socket	NR	NR	NR	participant/site as reported	extraction site/socket or participant as reported	4 months
Zampara et al. [8]	Randomized controlled clinical trial; four arms	32	32 extraction sites	45-70 years	NR	Maxilla and mandible	Anterior/posterior	NR	NR	NR	NR	participant/site as reported	extraction site/socket or participant as reported	NR in extracted text; biopsy at implant re-entry
Gokul et al. [9]	Prospective randomized clinical trial; two arms	20 analyzed (24 randomized)	20 mandibular posterior extraction sites	NR	Smokers excluded	Mandible	Posterior	NR	NR	NR	NR	participant/site as reported	extraction site/socket or participant as reported	6 months
Elfana et al.	Parallel RCT;	20	20 non-molar	31.2 -	NR	Maxilla and	Anterior/poster	NR	NR	NR	NR	participant/	extraction	4 months

[10]	two arms		extraction sites	33.5 years by group		mandible	ior					site as reported	on site/socket or participant as reported	
Abad et al. [11]	Parallel RCT; two arms	28	28 extraction sockets	NR	NR	NR	NR	NR	NR	NR	NR	participant/site as reported	extraction site/socket or participant as reported	4 months
Hussain et al. [12]	Parallel RCT; two arms	32 randomized; 29 completed/analyzed	32 maxillary anterior/non-molar sites randomized; 29 analyzed	NR	NR	Maxilla	Anterior	NR	NR	NR	NR	participant/site as reported	extraction site/socket or participant as reported	NR in extracted table; histology/radiography at re-entry
Clark et al. [13]	Parallel RCT; two arms	45 enrolled; 40 completed/analyzed	40 extraction sockets	NR	Tobacco users excluded	NR	NR	NR	NR	NR	NR	participant/site as reported	extraction site/socket or participant as reported	NR in extracted summary; biopsy at re-entry
Thakkar et al. [14]	Parallel clinical/radiographic randomized study; two arms	36 single-rooted teeth/sites; patient number NR	36 maxillary/mandibular single-rooted sockets	20-55 years	NR	NR	NR	NR	NR	NR	NR	participant/site as reported	extraction site/socket or participant as reported	NR in extracted summary
Schnutenhaus et al. [15]	Parallel RCT; two arms	31	NR	NR	NR	Maxilla	Anterior	NR	NR	NR	NR	participant/site as reported	extraction site/socket or participant as reported	8-11 weeks
Ouyyamwongs et al. [16]	Split-mouth RCT; two arms	12 subjects	40 extraction sites	20-22 years; mean 20.5 - 0.8 year	NR	Maxilla and mandible	Posterior	NR	NR	NR	NR	site/tooth within participant	extraction site/socket or participant as	2, 4, 6 and 8 weeks; main endpoint 8 weeks

				s									reported	
Dhamija et al. [17]	Parallel randomized study; two arms	20	20 extraction sockets	45.6 years; range 35-60 years	NR	NR	NR	NR	NR	NR	NR	participant/ site as reported	extraction site/ socket or participant as reported	6 months; implant placement around 7 months
Girish Kumar et al. [18]	Prospective randomized study; two arms	48	48 extraction sockets	NR	NR	Mandible	Posterior	NR	NR	NR	NR	participant/ site as reported	extraction site/ socket or participant as reported	6 months; broader follow-up to 18 months reported
Ivanova et al. [19]	Randomized controlled clinical study; three arms	28 analyzed (60 screened)	NR	NR	NR	NR	NR	NR	NR	NR	NR	participant/ site as reported	extraction site/ socket or participant as reported	4 months
Castro et al. [20]	Split-mouth RCT; two arms	21 included	60 teeth in premaxilla	NR	NR	Maxilla	NR	NR	NR	NR	NR	site/tooth within participant	extraction site/ socket or participant as reported	3 months
Demetter et al. [21]	Parallel RCT; three arms	66 enrolled; 59 completed; 58 clinical and 57 histologic analyses	66 non-molar extraction sites randomized; 59 completed	25-81 years; mean 55.5 years enrolled; mean 56.9 years completed	Included light smokers; group-level smoker counts reported	NR	Posterior	Defective/dehiscence socket	NR	NR	NR	participant/ site as reported	extraction site/ socket or participant as reported	18-20 weeks
Corning and Mealey [22]	Parallel RCT; two arms	44 enrolled; 38 completed; 37 analyzed for some clinical/ histologic	44 extraction sites randomized; 38 completed	22-84 years; mean 58 years enrolled; mea	>10 cigarettes/ day excluded	NR	NR	Defective/dehiscence socket	NR	NR	NR	participant/ site as reported	extraction site/ socket or participant as repo	12 weeks

		outcomes		n 59 years completed									rted	
Saito et al. [23]	Multicenter parallel RCT; two arms	45	NR	NR	NR	NR	NR	NR	NR	NR	NR	participant/site as reported	extraction site/socket or participant as reported	16-20 weeks; 12-month implant follow-up reported
Bajaj et al. [24]	Randomized controlled clinical trial; split-mouth/two grafts	20	40 mandibular molar extraction sites	NR	NR	Mandible	Posterior	NR	NR	NR	NR	site/tooth within participant	extraction site/socket or participant as reported	3-4 months/16 weeks
Shahdad et al. [25]	Parallel RCT; two arms	30	NR	NR	NR	Maxilla	Anterior	NR	NR	NR	NR	participant/site as reported	extraction site/socket or participant as reported	6 months/22 weeks
Choi et al. [26]	Multicenter split-mouth/within-patient RCT; two arms	38-39 reported	NR	NR	NR	NR	NR	NR	NR	NR	NR	site/tooth within participant	extraction site/socket or participant as reported	5 months
Nainoor et al. [27]	Split-mouth, double-blinded RCT	20	40 mandibular molar extraction sites	NR	NR	Mandible	Posterior	NR	NR	NR	NR	site/tooth within participant	extraction site/socket or participant as reported	4 months
Barone et al. [28]	Parallel RCT; two arms	40	40 extraction sites	26-69 years	NR	NR	NR	NR	NR	NR	NR	participant/site as reported	extraction site/socket or participant as reported	7 months
Nunes et al.	Split-mouth	15	30 upper anterior	39-65	NR	Maxilla	NR	NR	NR	NR	NR	site/tooth	extracti	6 months

[29]	RCT; two arms		extraction sockets	years								within participant	on site/socket or participant as reported	
Azangookhi avi et al. [30]	Parallel RCT; two arms	32 analyzed (35 enrolled)	32 non-molar extraction sockets	Mean 38 years	NR	NR	Posterior	NR	NR	NR	NR	participant/site as reported	extraction site/socket or participant as reported	12 weeks/3 months
Schnutenhaus et al. [31]	Parallel randomized clinical study; two arms	88 in collective analysis; subset for histology/implant stability	NR	49.6 years overall; subsets reported	NR	NR	NR	NR	NR	NR	NR	participant/site as reported	extraction site/socket or participant as reported	8 - 1 weeks CBCT; 11 - 1 weeks implant placement
Aladmawy et al. [32]	Pilot split-mouth RCT; two arms	8	16 sites	54.2 - 9.7 years	Non-smokers required	NR	Anterior/posterior	Defective/dehiscence socket	NR	NR	NR	site/tooth within participant	extraction site/socket or participant as reported	6 months
Wr-bel et al. [33]	Parallel randomized controlled trial; two arms	10	10 maxillary premolar extraction sites	18-65 years	Smoking excluded	Maxilla	Posterior	NR	NR	NR	NR	participant/site as reported	extraction site/socket or participant as reported	7-10 days and 6 months
Baronee et al. [34]	Restricted multicenter, single-blind, parallel RCT; two arms	28 analyzed (30 planned)	28 single-tooth extraction sites	NR	>10 cigarettes/day excluded; light smokers asked to stop	NR	NR	NR	NR	NR	NR	participant/site as reported	extraction site/socket or participant as reported	1 and 3 months

Table S4. Intervention details, treatment-node coding and outcome assessment

Study	Intervention and comparator	Standard NMA treatment node(s)	CNMA component	Scaffold material	PRF type	Membrane use	PRF used as membrane/barrier	Follow-up	Outcome measurement method	Outcomes

			coding							
Estrin et al. (2026) [4]	Sticky bone + e-PRF membrane variants vs sticky bone + collagen membrane	COL, PRF, PRF-M	See Table S5	Bone allograft mixed with PRF	e-PRF/solid PRF variants	Yes; collagen membrane or e-PRF membrane	Yes or partially	3 months; wound healing at 2 weeks	CBCT/radiographic	CBCT ridge width/height, soft tissue thickness, early wound healing
Alrayyes et al. (2023) [5]	A-PRF; A/S-PRF; FDBA + collagen membrane; resorbable collagen plug	NR	See Table S5	FDBA; factor-enriched graft matrix; collagen plug	A-PRF; A/S-PRF	Yes in FDBA/CM group; PRF used as biologic barrier in PRF groups	Yes or partially	6 months	CBCT/radiographic	CBCT ridge height/width; histology at implant placement
Talebi Ardakani et al. (2024) [6]	Allograft + collagen type I + I-PRF vs allograft + collagen type I	ALG+COL, ALG+PRF+COL	See Table S5	Allograft; collagen type I	I-PRF	Yes; collagen type I used in both groups	No/NR	3 months	CBCT/radiographic	CBCT ridge dimensions; clinical ridge width; histomorphometry
Gowda et al. (2023) [7]	Partially demineralized dentin matrix block + A-PRF+/i-PRF vs A-PRF+ plug	DT+PRF, PRF	See Table S5	Autologous tooth/dentin matrix	A-PRF+; i-PRF	PRF used as plug/barrier; no separate membrane reported	Yes or partially	4 months	CBCT/radiographic	Clinical and CBCT ridge height/width
Zampara et al. (2022) [8]	Allograft, xenograft, alloplast grafts and standard care	NR	See Table S5	Allograft; xenograft; alloplast	NR	Barrier membrane/GBR reported	No/NR	NR in extracted text; biopsy at implant re-entry	clinical/histologic	Clinical healing; histologic/histomorphometric outcomes
Gokul et al. (2025) [9]	Eggshell-derived hydroxyapatite vs DFDBA; both covered with PRF	NR	See Table S5	Eggshell-derived hydroxyapatite; DFDBA	PRF	PRF as barrier membrane	Yes or partially	6 months	CBCT/radiographic	Clinical and CBCT/radiographic ridge parameters
Elfana et al. (2021) [10]	Autogenous whole-tooth graft vs autogenous demineralized dentin graft	DT	See Table S5	Autogenous whole-tooth graft; autogenous demineralized dentin graft	NR	Collagen membrane/coverage reported	No/NR	4 months	CBCT/radiographic	CBCT dimensional change; histologic healing
Abad et al. (2023) [11]	L-PRF vs spontaneous healing	NH, PRF	See Table S5	No scaffold material in L-PRF arm	L-PRF	No separate membrane	No/NR	4 months	CBCT/radiographic	CBCT ridge dimensions, STL volumetric contour, patient-reported outcomes
Hussain et al. (2023) [12]	Autogenous dentin biomaterial vs comparator graft/control	DT, NH	See Table S5	Autogenous dentin biomaterial	NR	NR	No/NR	NR in extracted table; histology/radiography at re-entry	CBCT/radiographic	Clinical, tomographic/radiographic and histologic ARP outcomes
Clark et al. (2018) [13]	A-PRF + freeze-dried bone allograft vs control/allograft-based ARP	ALG, ALG+PRF, NH, PRF	See Table S5	Freeze-dried bone allograft	A-PRF	NR	No/NR	NR in extracted summary; biopsy at re-entry	NR	Histomorphometry, micro-CT bone density and ridge measurements
Thakkar	DFDBA +	ALG,	See	DFDBA	PRF	PRF used	Yes or	NR in	CBCT/radi	Clinical and

et al. (2016) [14]	PRF vs DFDBA alone	ALG+PRF	Table S5			adjunctively; no separate membrane reported	partially	extracted summary	ographic	radiographic bone fill/ridge dimensions
Schnutenhaus et al. (2018) [15]	Collagen cone/collagen material vs spontaneous healing/control	NR	See Table S5	Collagen material/cone	NR	Collagen material; membrane/cone procedure in related analysis	No/NR	8-11 weeks	CBCT/radiographic	CBCT/radiographic ridge preservation; clinical outcomes
Ouyyamwongs et al. (2019) [16]	Autologous demineralized tooth matrix + PRF vs PRF alone	DT+PRF, PRF	See Table S5	Autologous demineralized tooth matrix	PRF	PRF as socket coverage; no separate membrane reported	Yes or partially	2, 4, 6 and 8 weeks; main endpoint 8 weeks	CBCT/radiographic	Clinical/radiographic ridge width and height
Dhamija et al. (2020) [17]	DFDBA + PRF vs comparator ARP protocol	NR	See Table S5	DFDBA	PRF	NR	No/NR	6 months; implant placement around 7 months	clinical/histologic	Clinical, histologic and radiologic bone formation
Girish Kumar et al. (2018) [18]	PRF socket plug with bone substitute vs PRF socket plug alone	NR	See Table S5	Bone substitute in test arm	PRF	PRF plug; no separate membrane reported	Yes or partially	6 months; broader follow-up to 18 months reported	CBCT/radiographic	Clinical and radiographic socket healing/ridge preservation
Ivanova et al. (2019) [19]	A-PRF alone, A-PRF + allograft, allograft/control strategy	ALG, NH, PRF	See Table S5	Allograft	A-PRF	NR	No/NR	4 months	NR	Dimensional ridge alteration; histomorphometry
Castro et al. (2021) [20]	L-PRF vs A-PRF+ matrices	NH, PRF	See Table S5	No scaffold material	L-PRF; A-PRF+	PRF matrices; no separate membrane	Yes or partially	3 months	CBCT/radiographic	CBCT/radiographic dimensional change and bone structure
Demetter et al. (2017) [21]	Cortical FDBA vs cancellous FDBA vs combined cortico-cancellous FDBA	ALG	See Table S5	Cortical, cancellous, combined FDBA	NR	NR	No/NR	18-20 weeks	clinical/histologic	Histomorphometric vital bone/residual graft; clinical ridge dimensions
Corning and Mealey (2019) [22]	Mineralized FDBA vs mineralized solvent-dehydrated bone allograft	ALG	See Table S5	Mineralized FDBA; mineralized solvent-dehydrated allograft	NR	NR	No/NR	12 weeks	clinical/histologic	Clinical ridge changes and primary histologic outcomes
Saito et al. (2021) [23]	PLGA-coated --TCP vs comparator socket grafting protocol	AP+M, XG+M	See Table S5	PLGA-coated --TCP/alloplastic material	NR	NR	No/NR	16-20 weeks; 12-month implant follow-up reported	CBCT/radiographic	CBCT ridge dimensions; histology; implant-related outcomes
Bajaj et al. (2025) [24]	Nanocrystalline hydroxyapatite + DBM + T-PRF vs comparator graft protocol	NR	See Table S5	Nanocrystalline hydroxyapatite; DBM	T-PRF	T-PRF used as barrier/adjunct	Yes or partially	3-4 months/16 weeks	CBCT/radiographic	Clinical and radiographic ridge dimensions
Shahdad et al. (2020)	PEG-based synthetic membrane	NR	See Table S5	Bone substitute used in	NR	Yes; PEG-based synthetic	No/NR	6 months/22 weeks	CBCT/radiographic	CBCT/radiographic ridge preservation;

[25]	vs porcine-derived collagen membrane with bone substitute			ARP protocol		or porcine collagen membrane				clinical outcomes
Choi et al. (2025) [26]	Moldable bone material vs particulate bone material	NR	See Table S5	Moldable and particulate bone substitutes	NR	NR	No/NR	5 months	clinical/histologic	Radiologic and histologic ARP outcomes
Nainoor et al. (2026) [27]	Eggshell-derived nanohydroxyapatite vs DFDBA; both covered with PRF	NR	See Table S5	Eggshell-derived nanohydroxyapatite; DFDBA	PRF	PRF as barrier membrane	Yes or partially	4 months	CBCT/radiographic	Clinical and radiographic ridge dimensions
Barone et al. (2008) [28]	Corticocancellous porcine xenograft + collagen membrane vs extraction alone	NH, XG+M	See Table S5	Corticocancellous porcine xenograft	NR	Yes; collagen membrane in ridge-preservation arm	No/NR	7 months	clinical/histologic	Clinical ridge dimensions; histomorphometry
Nunes et al. (2018) [29]	Synthetic bone substitute + collagen membrane vs spontaneous healing/control	AP+M, COL	See Table S5	Synthetic bone substitute	NR	Yes; collagen membrane in test sites	No/NR	6 months	clinical/histologic	Clinical and cone-beam tomographic ridge dimensions
Azangookhiavi et al. (2020) [30]	FDBA vs PRF	ALG, PRF	See Table S5	FDBA	PRF	No separate membrane reported	No/NR	12 weeks/3 months	CBCT/radiographic	Clinical and radiographic ridge height/width
Schnutenhaus et al. (2020) [31]	Collagen cone + collagen membrane vs control/no ARP	NR	See Table S5	Collagen cone/material	NR	Yes; collagen cone + membrane procedure	No/NR	8 - 1 weeks CBCT; 11 - 1 weeks implant placement	CBCT/radiographic	CBCT bone density; histomorphometry; implant primary stability
Aladmawy et al. (2022) [32]	Allograft + nonresorbable membrane with primary closure vs without primary closure	NR	See Table S5	Freeze-dried bone allograft	NR	Yes; nonresorbable membrane	No/NR	6 months	NR	Histomorphometric new vital bone/residual graft/marrow tissue
Wr-bel et al. (2026) [33]	Allogeneic material + collagen membrane vs xenogeneic material + collagen membrane	NR	See Table S5	Allogeneic material; deproteinized bovine xenograft	NR	Yes; collagen membrane	No/NR	7-10 days and 6 months	CBCT/radiographic	Micro-CBCT ridge dimensions/bone density; histology
Barone et al. (2016) [34]	Collagenated cortico-cancellous porcine bone vs cortical porcine bone; both with	NH, XG+M	See Table S5	Porcine xenograft variants	NR	Yes; collagen membrane	No/NR	1 and 3 months	CBCT/radiographic	Scanned plaster casts/volumetric ridge remodeling; CBCT-based VOI described

collagen membrane

Table S5. Complete treatment-node and component-coding matrix

Treatment node	Complete intervention class	Components	Study occurrence, n	REF	ALG	XG	AP	COL	Tooth/dentin scaffold	PRF adjunct	MEM	Horizontal CNMA	Buccal CNMA
NH	Natural healing/blood clot	REF	6	1	0	0	0	0	0	0	0	Yes	Yes
COL	Collagen plug/cone/material	COL	3	0	0	0	0	1	0	0	0	Yes	Yes
PRF	PRF alone	PRF	8	0	0	0	0	0	0	1	0	Yes	Yes
PRF-M	PRF membrane/barrier	PRF + MEM	1	0	0	0	0	0	0	1	1	No	No
ALG	Allograft	ALG	8	0	1	0	0	0	0	0	0	Yes	Yes
ALG+PRF	Allograft + PRF	ALG + PRF	8	0	1	0	0	0	0	1	0	Yes	Yes
ALG+M	Allograft + membrane	ALG + MEM	5	0	1	0	0	0	0	0	1	Yes	Yes
ALG+PRF +M	Allograft + PRF + membrane	ALG + PRF + MEM	3	0	1	0	0	0	0	1	1	No	No
XG	Xenograft	XG	2	0	0	1	0	0	0	0	0	No	No
XG+M	Xenograft + membrane	XG + MEM	4	0	0	1	0	0	0	0	1	Yes	Yes
AP	Alloplast	AP	5	0	0	0	1	0	0	0	0	No	No
AP+M	Alloplast + membrane	AP + MEM	3	0	0	0	1	0	0	0	1	Yes	Yes
AP+PRF	Alloplast + PRF	AP + PRF	3	0	0	0	1	0	0	1	0	Yes	Yes
AP+PRF+ M	Alloplast + PRF + membrane	AP + PRF + MEM	2	0	0	0	1	0	0	1	1	No	No
DT	Tooth/dentin scaffold	DT	3	0	0	0	0	0	1	0	0	Yes	No
DT+PRF	Tooth/dentin scaffold + PRF	DT + PRF	3	0	0	0	0	0	1	1	0	Yes	Yes
MIX+PRF	Mixed scaffold + PRF	ALG + AP + PRF	1	0	1	0	1	0	0	1	0	No	No

Note: Component occurrence was summarized according to whether each component appeared as a standalone intervention or in combination with other components across the treatment network. PRF appeared alone in 8 and in combination in 21 intervention occurrences; allograft in 8 and 17, respectively; barrier membrane in 0 and 18; xenograft in 2 and 4; alloplast in 5 and 9; tooth/dentin-derived scaffold in 3 and 3; and collagen scaffold in 3 and 0. Because multi-arm trials could contribute more than one eligible intervention, these counts represent intervention occurrences rather than numbers of unique RCTs.

Table S6. Study-level Cochrane RoB 2 judgments

Study	Year	D1 Randomization	D2 Deviations	D3 Missing data	D4 Outcome measurement	D5 Selective reporting	Overall
Estrin et al. [4]	2026	Some concerns	Some concerns	Low	Some concerns	Some concerns	Some concerns
Alrayyes et al. [5]	2023	Some concerns	Some concerns	Low	Some concerns	Some concerns	Some concerns
Talebi Ardakani et al. [6]	2024	Low	Low	Low	Some concerns	Low	Some concerns
Gowda et al. [7]	2023	Some concerns	Some concerns	Low	Some concerns	Some concerns	Some concerns
Zampara et al. [8]	2022	Some concerns	Some concerns	Low	Some concerns	Some concerns	Some concerns
Gokul et al. [9]	2025	Some concerns	Some concerns	Some concerns	Some concerns	Some concerns	Some concerns
Elfana et al. [10]	2021	Some concerns	Some concerns	Low	Some concerns	Some concerns	Some concerns
Abad et al. [11]	2023	Some concerns	Some concerns	Low	Some concerns	Low	Some concerns
Hussain et al. [12]	2023	Some concerns	Some concerns	Some concerns	Some concerns	Some concerns	Some concerns

Clark et al. [13]	2018	Low	Some concerns	Some concerns	Low	Low	Some concerns
Thakkar et al. [14]	2016	Some concerns	Some concerns	Low	Some concerns	Some concerns	Some concerns
Schnutenhaus et al. [15]	2018	Low	Low	Low	Low	Low	Low
Ouyyamwongs et al. [16]	2019	Some concerns	Some concerns	Low	High	Some concerns	High
Dhamija et al. [17]	2020	Some concerns	Some concerns	Low	Some concerns	Some concerns	Some concerns
Girish Kumar et al. [18]	2018	Some concerns	Some concerns	Low	Some concerns	Some concerns	Some concerns
Ivanova et al. [19]	2019	Some concerns	Some concerns	Some concerns	Some concerns	Some concerns	Some concerns
Castro et al. [20]	2021	Low	Some concerns	Low	Some concerns	Low	Some concerns
Demetter et al. [21]	2017	Low	Some concerns	Some concerns	Low	Low	Some concerns
Corning and Mealey [22]	2019	Low	Low	Some concerns	Low	Low	Some concerns
Saito et al. [23]	2021	Low	Low	Low	Low	Low	Low
Bajaj et al. [24]	2024	Some concerns	Some concerns	Low	High	Some concerns	High
Shahdad et al. [25]	2020	Some concerns	Some concerns	Low	Some concerns	Some concerns	Some concerns
Choi et al. [26]	2025	Low	Some concerns	Low	Some concerns	Low	Some concerns
Nainoor et al. [27]	2026	Low	Low	Low	Some concerns	Low	Some concerns
Barone et al. [28]	2008	Low	Low	Low	Low	Low	Low
Nunes et al. [29]	2018	Some concerns	Some concerns	Low	High	Some concerns	High
Azangookhiavi et al. [30]	2020	Low	Low	Low	Low	Low	Low
Schnutenhaus et al. [31]	2020	Low	Low	Low	Low	Low	Low
Aladmawy et al. [32]	2022	High	Some concerns	Low	High	Some concerns	High
Wrobel et al. [33]	2026	Some concerns	High	High	Some concerns	Some concerns	High
Barone et al. [34]	2016	Low	Low	Low	Low	Low	Low

Table S7. Pairwise meta-analysis results for direct comparisons with at least two studies

Outcome	Direct comparison	No. of studies	Studies included	MD (mm)	95% CI	tau-squared	I-squared (%)	95% prediction interval	Interpretation	Statistical model	Effect direction	Split-mouth correlation assumption
Horizontal ridge-width change	Allograft + PRF vs allograft	2	10, 11	-0.61	-0.98 to -0.24	0	0	NR	Favours first treatment	Random-effects model	Negative MD favours first treatment listed	Paired-design sensitivity addressed narratively where applicable
Horizontal ridge-width change	PRF vs natural healing/blood clot	5	08, 10, 15, 16, 17	-0.29	-0.84 to 0.26	0.243	77.9	-2.10 to 1.52	No statistically significant difference	Random-effects model	Negative MD favours first treatment listed	Paired-design sensitivity addressed narratively where applicable
Horizontal ridge-width change	PRF alone vs allograft	3	10, 16, 27	0.2	0.05 to 0.36	0	0	-0.79 to 1.20	Favours comparator	Random-effects model	Negative MD favours first treatment listed	Paired-design sensitivity addressed

												narratively where applicable
Horizontal ridge-width change	Allograft vs natural healing/blood clot	2	10, 16	-1.03	-1.30 to -0.75	0.005	2.3	NR	Favours first treatment	Random-effects model	Negative MD favours first treatment listed	Paired-design sensitivity addressed narratively where applicable
Buccal vertical ridge-height change	Allograft + PRF vs allograft	2	10, 11	-0.33	-0.63 to -0.03	0	0	NR	Favours first treatment	Random-effects model	Negative MD favours first treatment listed	Paired-design sensitivity addressed narratively where applicable
Buccal vertical ridge-height change	PRF vs natural healing/blood clot	5	08, 10, 15, 16, 17	-0.31	-0.73 to 0.11	0.127	72.9	-1.63 to 1.02	No statistically significant difference	Random-effects model	Negative MD favours first treatment listed	Paired-design sensitivity addressed narratively where applicable
Buccal vertical ridge-height change	PRF alone vs allograft	3	10, 16, 27	0.04	-0.08 to 0.17	0	0	-0.76 to 0.85	No statistically significant difference	Random-effects model	Negative MD favours first treatment listed	Paired-design sensitivity addressed narratively where applicable
Buccal vertical ridge-height change	Allograft vs natural healing/blood clot	2	10, 16	-0.72	-1.57 to 0.12	0.23	38.8	NR	No statistically significant difference	Random-effects model	Negative MD favours first treatment listed	Paired-design sensitivity addressed narratively where applicable

Note: MD, mean difference; CI, confidence interval; NE, not estimable. Negative MDs indicate less dimensional loss for the first-listed treatment relative to the comparator and therefore favor the first-listed treatment. Random-effects models were used for all comparisons, with prediction intervals reported where estimable..

Table S8. Full league table for horizontal ridge-width change

Treatment	ALG	ALG+M	ALG+PRF	AP+M	AP+PRF	COL	DT	DT+PRF	NH	PRF	XG+M	XG+PRF
ALG	.	1.01 (-0.36 to 2.37)	0.50 (0.03 to 0.97)	0.68 (-0.35 to 1.70)	-0.48 (-1.26 to 0.30)	-0.31 (-1.15 to 0.53)	1.30 (0.09 to 2.50)	0.75 (-0.03 to 1.53)	-0.77 (-1.28 to -0.27)	-0.41 (-0.87 to 0.05)	1.01 (0.10 to 1.92)	0.16 (-0.95 to 1.27)
ALG+M	-1.01 (-2.37 to 0.36)	.	-0.50 (-1.93 to 0.92)	-0.33 (-1.69 to 1.03)	-1.49 (-2.92 to -0.05)	-1.32 (-2.71 to 0.07)	0.29 (-1.40 to 1.98)	-0.26 (-1.72 to 1.20)	-1.78 (-3.06 to -0.50)	-1.42 (-2.73 to -0.10)	0.00 (-1.01 to 1.01)	-0.85 (-2.48 to 0.79)
ALG+PRF	-0.50 (-0.97 to -0.03)	0.50 (-0.92 to 1.93)	.	0.18 (-0.93 to 1.28)	-0.98 (-1.86 to -0.10)	-0.81 (-1.75 to 0.12)	0.79 (-0.48 to 2.07)	0.25 (-0.63 to 1.13)	-1.28 (-1.93 to -0.63)	-0.91 (-1.53 to -0.29)	0.50 (-0.50 to 1.50)	-0.34 (-1.52 to 0.84)
AP+M	-0.68 (-1.70 to 0.35)	0.33 (-1.03 to 1.69)	-0.18 (-1.28 to 0.93)	.	-1.16 (-2.28 to -0.03)	-0.99 (-1.80 to -0.18)	0.62 (-0.83 to 2.07)	0.07 (-1.06 to 1.20)	-1.45 (-2.40 to -0.50)	-1.09 (-2.03 to -0.15)	0.33 (-0.58 to 1.24)	-0.52 (-1.89 to 0.85)
AP+PRF	0.48 (-	1.49	0.98 (0.10	1.16	.	0.17 (-	1.78	1.23	-0.29	0.07 (-	1.49	0.64 (-

	0.30 to 1.26)	(0.05 to 2.92)	to 1.86)	(0.03 to 2.28)		0.80 to 1.13)	(0.49 to 3.06)	(0.32 to 2.14)	(-0.97 to 0.38)	0.59 to 0.73)	(0.47 to 2.50)	0.15 to 1.43)
COL	0.31 (-0.53 to 1.15)	1.32 (-0.07 to 2.71)	0.81 (-0.12 to 1.75)	0.99 (0.18 to 1.80)	-0.17 (-1.13 to 0.80)	.	1.61 (0.26 to 2.96)	1.06 (0.11 to 2.01)	-0.46 (-1.24 to 0.32)	-0.10 (-0.81 to 0.62)	1.32 (0.37 to 2.27)	0.47 (-0.77 to 1.72)
DT	-1.30 (-2.50 to -0.09)	-0.29 (-1.98 to 1.40)	-0.79 (-2.07 to 0.48)	-0.62 (-2.07 to 0.83)	-1.78 (-3.06 to -0.49)	-1.61 (-2.96 to -0.26)	.	-0.55 (-1.87 to 0.78)	-2.07 (-3.17 to -0.97)	-1.71 (-2.87 to -0.54)	-0.29 (-1.64 to 1.06)	-1.14 (-2.64 to 0.37)
DT+PRF	-0.75 (-1.53 to 0.03)	0.26 (-1.20 to 1.72)	-0.25 (-1.13 to 0.63)	-0.07 (-1.20 to 1.06)	-1.23 (-2.14 to -0.32)	-1.06 (-2.01 to -0.11)	0.55 (-0.78 to 1.87)	.	-1.52 (-2.26 to -0.78)	-1.16 (-1.79 to -0.53)	0.26 (-0.79 to 1.30)	-0.59 (-1.79 to 0.62)
NH	0.77 (0.27 to 1.28)	1.78 (0.50 to 3.06)	1.28 (0.63 to 1.93)	1.45 (0.50 to 2.40)	0.29 (-0.38 to 0.97)	0.46 (-0.32 to 1.24)	2.07 (0.97 to 3.17)	1.52 (0.78 to 2.26)	.	0.36 (-0.03 to 0.75)	1.78 (0.99 to 2.57)	0.93 (-0.10 to 1.97)
PRF	0.41 (-0.05 to 0.87)	1.42 (0.10 to 2.73)	0.91 (0.29 to 1.53)	1.09 (0.15 to 2.03)	-0.07 (-0.73 to 0.59)	0.10 (-0.62 to 0.81)	1.71 (0.54 to 2.87)	1.16 (0.53 to 1.79)	-0.36 (-0.75 to 0.03)	.	1.42 (0.58 to 2.26)	0.57 (-0.46 to 1.60)
XG+M	-1.01 (-1.92 to -0.10)	-0.00 (-1.01 to 1.01)	-0.50 (-1.50 to 0.50)	-0.33 (-1.24 to 0.58)	-1.49 (-2.50 to -0.47)	-1.32 (-2.27 to -0.37)	0.29 (-1.06 to 1.64)	-0.26 (-1.30 to 0.79)	-1.78 (-2.57 to -0.99)	-1.42 (-2.26 to -0.58)	.	-0.85 (-2.13 to 0.44)
XG+PRF	-0.16 (-1.27 to 0.95)	0.85 (-0.79 to 2.48)	0.34 (-0.84 to 1.52)	0.52 (-0.85 to 1.89)	-0.64 (-1.43 to 0.15)	-0.47 (-1.72 to 0.77)	1.14 (-0.37 to 2.64)	0.59 (-0.62 to 1.79)	-0.93 (-1.97 to 0.10)	-0.57 (-1.60 to 0.46)	0.85 (-0.44 to 2.13)	.

Table S9. Full league table for buccal vertical ridge-height change

Treatment	ALG	ALG+M	ALG+PRF	AP+M	AP+PRF	COL	DT+PRF	NH	PRF	XG+M
ALG	.	2.22 (0.23 to 4.21)	0.46 (-0.36 to 1.27)	0.84 (-0.80 to 2.48)	-0.21 (-1.54 to 1.12)	0.08 (-1.30 to 1.46)	1.68 (0.22 to 3.14)	-0.81 (-1.67 to 0.05)	-0.31 (-1.12 to 0.50)	1.42 (-0.09 to 2.94)
ALG+M	-2.22 (-4.21 to -0.23)	.	-1.77 (-3.88 to 0.35)	-1.38 (-3.19 to 0.42)	-2.43 (-4.55 to -0.31)	-2.14 (-4.10 to -0.18)	-0.54 (-2.78 to 1.69)	-3.03 (-4.86 to -1.20)	-2.53 (-4.41 to -0.66)	-0.80 (-2.09 to 0.49)
ALG+PRF	-0.46 (-1.27 to 0.36)	1.77 (-0.35 to 3.88)	.	0.38 (-1.40 to 2.17)	-0.67 (-2.18 to 0.85)	-0.38 (-1.94 to 1.19)	1.22 (-0.41 to 2.86)	-1.26 (-2.38 to -0.14)	-0.77 (-1.86 to 0.33)	0.97 (-0.71 to 2.65)
AP+M	-0.84 (-2.48 to 0.80)	1.38 (-0.42 to 3.19)	-0.38 (-2.17 to 1.40)	.	-1.05 (-2.85 to 0.75)	-0.76 (-2.06 to 0.54)	0.84 (-1.07 to 2.75)	-1.65 (-3.12 to -0.17)	-1.15 (-2.62 to 0.32)	0.58 (-0.68 to 1.85)
AP+PRF	0.21 (-1.12 to 1.54)	2.43 (0.31 to 4.55)	0.67 (-0.85 to 2.18)	1.05 (-0.75 to 2.85)	.	0.29 (-1.29 to 1.87)	1.89 (0.24 to 3.54)	-0.60 (-1.72 to 0.52)	-0.10 (-1.22 to 1.02)	1.63 (-0.05 to 3.32)
COL	-0.08 (-1.46 to 1.30)	2.14 (0.18 to 4.10)	0.38 (-1.19 to 1.94)	0.76 (-0.54 to 2.06)	-0.29 (-1.87 to 1.29)	.	1.60 (-0.07 to 3.27)	-0.89 (-2.13 to 0.36)	-0.39 (-1.53 to 0.76)	1.34 (-0.13 to 2.82)
DT+PRF	-1.68 (-3.14 to -0.22)	0.54 (-1.69 to 2.78)	-1.22 (-2.86 to 0.41)	-0.84 (-2.75 to 1.07)	-1.89 (-3.54 to -0.24)	-1.60 (-3.27 to 0.07)	.	-2.49 (-3.85 to -1.12)	-1.99 (-3.20 to -0.78)	-0.26 (-2.08 to 1.57)
NH	0.81 (-0.05 to 1.67)	3.03 (1.20 to 4.86)	1.26 (0.14 to 2.38)	1.65 (0.17 to 3.12)	0.60 (-0.52 to 1.72)	0.89 (-0.36 to 2.13)	2.49 (1.12 to 3.85)	.	0.50 (-0.13 to 1.12)	2.23 (0.93 to 3.53)
PRF	0.31 (-0.50 to 1.12)	2.53 (0.66 to 4.41)	0.77 (-0.33 to 1.86)	1.15 (-0.32 to 2.62)	0.10 (-1.02 to 1.22)	0.39 (-0.76 to 1.53)	1.99 (0.78 to 3.20)	-0.50 (-1.12 to 0.13)	.	1.73 (0.37 to 3.10)
XG+M	-1.42 (-2.94 to 0.09)	0.80 (-0.49 to 2.09)	-0.97 (-2.65 to 0.71)	-0.58 (-1.85 to 0.68)	-1.63 (-3.32 to 0.05)	-1.34 (-2.82 to 0.13)	0.26 (-1.57 to 2.08)	-2.23 (-3.53 to -0.93)	-1.73 (-3.10 to -0.37)	.

Table S10. Treatment rankings for the two primary outcomes

Outcome	Treatment node	Treatment	Studies	Participants/sites	P-score	Rank	Effect vs natural healing (95% CI)
Horizontal ridge-width change	DT	Dentin graft	1	14	0.879	1	-2.07 (-3.17 to -0.97)

Horizontal ridge-width change	XG+M	Xenograft + membrane	3	45	0.818	2	-1.78 (-2.57 to -0.99)
Horizontal ridge-width change	ALG+M	Allograft + membrane	1	5	0.785	3	-1.78 (-3.06 to -0.50)
Horizontal ridge-width change	DT+PRF	Dentin graft + PRF	1	8	0.721	4	-1.52 (-2.26 to -0.78)
Horizontal ridge-width change	AP+M	Alloplast + membrane	2	38	0.678	5	-1.45 (-2.40 to -0.50)
Horizontal ridge-width change	ALG+PRF	Allograft + PRF	3	34	0.62	6	-1.28 (-1.93 to -0.63)
Horizontal ridge-width change	XG+PRF	Xenograft + PRF	1	10	0.469	7	-0.93 (-1.97 to 0.10)
Horizontal ridge-width change	ALG	Allograft	5	78	0.386	8	-0.77 (-1.28 to -0.27)
Horizontal ridge-width change	COL	Collagen scaffold	2	26	0.244	9	-0.46 (-1.24 to 0.32)
Horizontal ridge-width change	PRF	PRF alone or PRF-based adjunct	8	185	0.196	10	-0.36 (-0.75 to 0.03)
Horizontal ridge-width change	AP+PRF	Alloplast + PRF	2	40	0.166	11	-0.29 (-0.97 to 0.38)
Horizontal ridge-width change	NH	Natural healing / blood clot	7	120	0.0361	12	Reference
Buccal vertical ridge-height change	ALG+M	Allograft + membrane	1	5	0.934	1	-3.03 (-4.86 to -1.20)
Buccal vertical ridge-height change	DT+PRF	Dentin graft + PRF	1	8	0.845	2	-2.49 (-3.85 to -1.12)
Buccal vertical ridge-height change	XG+M	Xenograft + membrane	3	45	0.787	3	-2.23 (-3.53 to -0.93)
Buccal vertical ridge-height change	AP+M	Alloplast + membrane	2	38	0.624	4	-1.65 (-3.12 to -0.17)
Buccal vertical ridge-height change	ALG+PRF	Allograft + PRF	3	34	0.538	5	-1.26 (-2.38 to -0.14)
Buccal vertical ridge-height change	COL	Collagen scaffold	2	26	0.376	6	-0.89 (-2.13 to 0.36)
Buccal vertical ridge-height change	ALG	Allograft	5	78	0.352	7	-0.81 (-1.67 to 0.05)
Buccal vertical ridge-height change	AP+PRF	Alloplast + PRF	1	30	0.281	8	-0.60 (-1.72 to 0.52)
Buccal vertical ridge-height change	PRF	PRF alone or PRF-based adjunct	8	185	0.223	9	-0.50 (-1.12 to 0.13)
Buccal vertical ridge-height change	NH	Natural healing / blood clot	6	105	0.039	10	Reference

Note: MD, mean difference; CI, confidence interval. Negative MDs indicate less dimensional loss relative to natural healing/blood clot healing and therefore favor better ridge preservation. Treatment rankings are based on P-scores from the random-effects network meta-analysis. P-scores should be interpreted cautiously and in conjunction with the corresponding effect estimates, 95% CIs, and the amount of supporting evidence, particularly for treatment nodes informed by few studies.

Table S11. Additive CNMA component estimates for primary dimensional outcomes

Outcome	component	component_label	MD (95% CI)	p_value
Horizontal ridge-width change	DT	Dentin graft	-1.35 (-1.82 to -0.87)	2.57e-08
Horizontal ridge-width	M	Barrier membrane	-1.31 (-2.18 to -0.44)	0.00303

change				
Horizontal ridge-width change	ALG	Allograft	-0.77 (-1.16 to -0.38)	0.000102
Horizontal ridge-width change	XG	Xenograft	-0.57 (-1.20 to 0.06)	0.0776
Horizontal ridge-width change	COL	Collagen scaffold	-0.42 (-1.07 to 0.23)	0.208
Horizontal ridge-width change	PRF	PRF adjunct	-0.40 (-0.66 to -0.14)	0.00271
Horizontal ridge-width change	AP	Alloplast	0.01 (-0.51 to 0.53)	0.966
Buccal vertical ridge-height change	DT	Dentin graft	-1.99 (-2.64 to -1.34)	2.16e-09
Buccal vertical ridge-height change	M	Barrier membrane	-1.79 (-2.82 to -0.76)	0.000669
Buccal vertical ridge-height change	COL	Collagen scaffold	-0.76 (-1.48 to -0.03)	0.0404
Buccal vertical ridge-height change	ALG	Allograft	-0.75 (-1.22 to -0.28)	0.00165
Buccal vertical ridge-height change	PRF	PRF adjunct	-0.44 (-0.73 to -0.14)	0.00384
Buccal vertical ridge-height change	XG	Xenograft	-0.23 (-0.93 to 0.48)	0.529
Buccal vertical ridge-height change	AP	Alloplast	0.04 (-0.52 to 0.60)	0.896

Table S12. Interaction CNMA estimates and identifiability

Interaction term	Estimate (MD, mm)	95% CI	P value	Identifiable	Supporting evidence	Model decision
XG x PRF	0.28	-1.21 to 1.76	0.716	Yes	Sparse XG/PRF component crossing	Not retained
AP x PRF	0.6	-0.97 to 2.18	0.453	Yes	Sparse AP/PRF component crossing	Not retained
COL x PRF	NA	NA	NA	No	No informative collagen scaffold plus PRF combination	Not estimable
MEM x PRF	NA	NA	NA	No	Sparse and partially collinear PRF-membrane information	Not estimable
Scaffold x MEM	NA	NA	NA	No	Membrane not sufficiently crossed with every scaffold type	Not estimable

Table S13. Model-fit comparison for horizontal ridge-width change

Model	No. of parameters	Approximate AIC	Residual deviance/Q	df	tau-squared	I-squared (%)	Preferred	Reason
Standard NMA	11	42.4	20.4	9	0.101	56	No	Complete intervention model
Additive CNMA	7	39.2	25.2	13	0.072	48.5	Yes	Lower approximate AIC; interpretable component effects
Interaction CNMA	9	42.3	24.3	11	0.091	54.8	No	Interaction estimates imprecise

Table S14A. Global inconsistency and heterogeneity analyses

outcome	tau2	tau	I2	Q_total	df_total	p_total	Q_within	df_within	p_within	Q_between	df_between	p_between

Horizontal ridge-width change	0.101	0.318	0.56	20.4	9	0.0154	0.541	2	0.763	19.9	7	0.00579
Buccal vertical ridge-height change	0.382	0.618	0.675	27.7	9	0.00109	2.62	2	0.269	25	7	0.000751

Table S14B. Node-splitting results for local inconsistency

outcome	comparison	direct_studies	direct_proportion	nma_MD	nma_lower	nma_upper	direct_MD	direct_lower	direct_upper	indirect_MD	indirect_lower	indirect_upper	diff_MD	diff_lower	diff_upper	z	p_value
Horizontal ridge-width change	ALG:ALG+PRF	3	0.948	0.503	0.0328	0.973	0.526	0.0429	1.01	0.0888	-1.97	2.14	0.437	-1.67	2.55	0.405	0.685
Horizontal ridge-width change	ALG:NH	2	0.701	-0.773	-1.28	-0.268	-0.937	-1.54	-0.333	-0.39	-1.31	0.534	-0.548	-1.65	0.556	-0.972	0.331
Horizontal ridge-width change	ALG:PRF	3	0.848	-0.411	-0.874	0.0532	-0.294	-0.798	0.209	-1.06	-2.25	0.129	0.769	-0.525	2.06	1.16	0.244
Horizontal ridge-width change	ALG+M:XG+M	1	1	0	-1.01	1.01	0	-1.01	1.01	NR	NR	NR	NR	NR	NR	NR	NR
Horizontal ridge-width change	ALG+PRF:NH	1	0.216	-1.28	-1.93	-0.625	-1	-2.4	0.401	-1.35	-2.09	-0.617	0.352	-1.23	1.93	0.436	0.663
Horizontal ridge-width change	ALG+PRF:PRF	1	0.273	-0.913	-1.53	-0.294	-0.9	-2.09	0.286	-0.918	-1.64	-0.192	0.0182	-1.37	1.41	0.0256	0.98
Horizontal ridge-width change	AP+M:COL	1	0.766	-0.99	-1.8	-0.178	-0.75	-1.68	0.178	-1.77	-3.45	-0.0965	1.02	-0.893	2.94	1.05	0.295
Horizontal ridge-width change	AP+M:XG+M	1	0.659	0.329	-0.58	1.24	-0.02	-1.14	1.1	1	-0.552	2.56	-1.02	-2.94	0.893	-1.05	0.295
Horizontal ridge-width change	AP+PRF:NH	1	0.808	-0.294	-0.965	0.378	-0.1	-0.847	0.647	-1.11	-2.64	0.424	1.01	-0.697	2.71	1.16	0.247
Horizontal ridge-width change	AP+PRF:PRF	1	0.843	0.0692	-0.595	0.733	-0.1	-0.823	0.623	0.979	-0.697	2.66	-1.08	-2.9	0.747	-1.16	0.247
Horizontal ridge-width change	AP+PRF:XG+PRF	1	0	0.64	-0.146	1.43	0.64	-0.146	1.43	NR	NR	NR	NR	NR	NR	NR	NR
Horizontal ridge-width	COL:PRF	1	0.833	-0.0983	-0.813	0.616	0.0725	-0.71	0.855	-0.951	-2.7	0.799	1.02	-0.893	2.94	1.05	0.295

change																	
Horizontal ridge-width change	DT:NH	1	1	-2.07	-3.17	-0.973	-2.07	-3.17	-0.973	NR	NR	NR	NR	NR	NR	NR	NR
Horizontal ridge-width change	DT+PRF:PRF	1	1	-1.16	-1.79	-0.533	-1.16	-1.79	-0.533	NR	NR	NR	NR	NR	NR	NR	NR
Horizontal ridge-width change	PRF:NH	5	0.936	-0.363	-0.752	0.0266	-0.326	-0.729	0.0765	-0.901	-2.44	0.639	0.574	-1.02	2.17	0.707	0.479
Horizontal ridge-width change	XG+M:NH	1	0.786	-1.78	-2.57	-0.994	-2	-2.89	-1.11	-0.976	-2.68	0.723	-1.02	-2.94	0.893	-1.05	0.295
Buccal vertical ridge-height change	ALG:ALG+PRF	3	0.963	0.456	-0.36	1.27	0.409	-0.422	1.24	1.67	-2.57	5.92	-1.27	-5.59	3.06	-0.574	0.566
Buccal vertical ridge-height change	ALG:NH	2	0.675	-0.806	-1.67	0.0548	-0.792	-1.84	0.256	-0.837	-2.35	0.675	0.0455	-1.79	1.89	0.0485	0.961
Buccal vertical ridge-height change	ALG:PRF	3	0.832	-0.31	-1.12	0.504	-0.11	-1	0.782	-1.3	-3.28	0.689	1.19	-0.991	3.36	1.07	0.285
Buccal vertical ridge-height change	ALG+M:XG+M	1	1	-0.8	-2.09	0.489	-0.8	-2.09	0.489	NR	NR	NR	NR	NR	NR	NR	NR
Buccal vertical ridge-height change	ALG+PRF:NH	1	0.25	-1.26	-2.38	-0.14	-2.8	-5.04	-0.556	-0.749	-2.05	0.546	-2.05	-4.64	0.541	-1.55	0.121
Buccal vertical ridge-height change	ALG+PRF:PRF	1	0.229	-0.765	-1.86	0.326	-0.8	-3.08	1.48	-0.755	-2	0.488	-0.0451	-2.64	2.55	-0.034	0.973
Buccal vertical ridge-height change	AP+M:COL	1	0.735	-0.76	-2.06	0.544	-0.08	-1.6	1.44	-2.65	-5.18	-0.113	2.57	-0.389	5.52	1.7	0.0887
Buccal vertical ridge-height change	AP+M:XG+M	1	0.757	0.584	-0.683	1.85	-0.04	-1.5	1.42	2.53	-0.044	5.09	-2.57	-5.52	0.389	-1.7	0.0887
Buccal vertical ridge-height change	AP+PRF:NH	1	0.812	-0.596	-1.72	0.523	-0.5	-1.74	0.742	-1.01	-3.6	1.57	0.513	-2.35	3.38	0.351	0.726
Buccal vertical ridge-height change	AP+PRF:PRF	1	0.8	-0.0995	-1.22	1.02	-0.2	-1.46	1.06	0.302	-2.21	2.81	-0.502	-3.31	2.31	-0.351	0.726
Buccal vertical ridge-height change	COL:PRF	1	0.816	-0.39	-1.53	0.755	0.0825	-1.18	1.35	-2.48	-5.15	0.185	2.57	-0.389	5.52	1.7	0.0887
Buccal vertical ridge-	DT+PRF:	1	1	-1.99	-3.2	-0.776	-1.99	-3.2	-0.776	NR	NR	NR	NR	NR	NR	NR	NR

height change	PRF																
Buccal vertical ridge-height change	PRF:NH	5	0.926	-0.497	-1.12	0.126	-0.392	-1.04	0.256	-1.8	-4.08	0.482	1.41	-0.964	3.78	1.16	0.244
Buccal vertical ridge-height change	XG+M:NH	1	0.739	-2.23	-3.53	-0.933	-2.9	-4.41	-1.39	-0.335	-2.87	2.2	-2.57	-5.52	0.389	-1.7	0.0887

Table S15. Sensitivity analyses for additive CNMA

Analysis	Studies	Arms	Components	MEM MD	MEM 95% CI	ALG MD	ALG 95% CI	PRF MD	PRF 95% CI	tau2	I2	Conclusion
Primary analysis	16	36	7	-1.31	-2.18 to -0.44	-0.773	-1.16 to -0.38	-0.402	-0.66 to -0.14	0.0724	0.485	Reference analysis
Excluding preliminary high-risk-of-bias studies	15	34	7	-1.47	-2.55 to -0.39	-0.758	-1.17 to -0.35	-0.382	-0.66 to -0.10	0.0827	0.521	No reversal of component hierarchy
Restricted to 4-6-month follow-up window	8	18	5	NR	NA to NA	-0.852	-1.53 to -0.17	-0.478	-0.88 to -0.08	0.136	0.73	No reversal of component hierarchy
Fixed-effect additive CNMA	16	36	7	-1.37	-1.99 to -0.74	-0.778	-0.96 to -0.60	-0.501	-0.66 to -0.35	NR	NR	No reversal of component hierarchy

Table S16. CINeMA/GRADE-informed certainty assessment for key standard NMA comparisons

Outcome	Comparison	MD (95% CI)	Studies	Participants/sites	Within-study bias	Imprecision	Heterogeneity	Incoherence	Overall certainty
Horizontal ridge-width change	Dentin graft vs natural healing	-2.07 (-3.17 to -0.97)	1	14	Serious	Not serious	Serious	No local incoherence; global inconsistency detected	Low
Horizontal ridge-width change	Xenograft + membrane vs natural healing	-1.78 (-2.57 to -0.99)	3	45	Serious	Not serious	Serious	No local incoherence; global inconsistency detected	Low
Horizontal ridge-width change	Allograft + membrane vs natural healing	-1.78 (-3.06 to -0.50)	1	5	Serious	Not serious	Serious	No local incoherence; global inconsistency detected	Low
Horizontal ridge-width change	Dentin graft + PRF vs natural healing	-1.52 (-2.26 to -0.78)	1	8	Serious	Not serious	Serious	No local incoherence; global inconsistency detected	Low
Horizontal ridge-width change	Alloplast + membrane vs natural healing	-1.45 (-2.40 to -0.50)	2	38	Serious	Not serious	Serious	No local incoherence; global inconsistency detected	Low
Horizontal ridge-width change	Allograft + PRF vs natural healing	-1.28 (-1.93 to -0.63)	3	34	Serious	Not serious	Serious	No local incoherence; global inconsistency detected	Low
Horizontal	Xenograft +	-0.93 (-	1	10	Serious	Serious	Serious	No local	Very low

ridge-width change	PRF vs natural healing	1.97 to 0.10)						incoherence; global inconsistency detected	
Horizontal ridge-width change	Allograft vs natural healing	-0.77 (-1.28 to -0.27)	5	78	Serious	Not serious	Serious	No local incoherence; global inconsistency detected	Low
Buccal vertical ridge-height change	Allograft + membrane vs natural healing	-3.03 (-4.86 to -1.20)	1	5	Serious	Not serious	Serious	No local incoherence; global inconsistency detected	Low
Buccal vertical ridge-height change	Dentin graft + PRF vs natural healing	-2.49 (-3.85 to -1.12)	1	8	Serious	Not serious	Serious	No local incoherence; global inconsistency detected	Low
Buccal vertical ridge-height change	Xenograft + membrane vs natural healing	-2.23 (-3.53 to -0.93)	3	45	Serious	Not serious	Serious	No local incoherence; global inconsistency detected	Low
Buccal vertical ridge-height change	Alloplast + membrane vs natural healing	-1.65 (-3.12 to -0.17)	2	38	Serious	Not serious	Serious	No local incoherence; global inconsistency detected	Low
Buccal vertical ridge-height change	Allograft + PRF vs natural healing	-1.26 (-2.38 to -0.14)	3	34	Serious	Not serious	Serious	No local incoherence; global inconsistency detected	Low
Buccal vertical ridge-height change	Collagen scaffold vs natural healing	-0.89 (-2.13 to 0.36)	2	26	Serious	Serious	Serious	No local incoherence; global inconsistency detected	Very low
Buccal vertical ridge-height change	Allograft vs natural healing	-0.81 (-1.67 to 0.05)	5	78	Serious	Serious	Serious	No local incoherence; global inconsistency detected	Very low
Buccal vertical ridge-height change	Alloplast + PRF vs natural healing	-0.60 (-1.72 to 0.52)	1	30	Serious	Serious	Serious	No local incoherence; global inconsistency detected	Very low

Table S17. Narrative certainty assessment for CNMA component effects

Component	MD (95% CI)	Evidence support and distribution	Identifiability	Precision	Heterogeneity/inconsistency	Model dependence	Risk of bias	Overall certainty
Dentin graft	-1.35 (-1.82 to -0.87)	Limited and unevenly distributed across tooth/dentin scaffold contrasts	Identifiable	Not serious	Serious: global network inconsistency	Some concern: model checks were performed at network level rather than component-specific level	Serious: contributing evidence included non-low risk-of-bias judgments	Low
Barrier membrane	-1.31 (-2.18 to -0.44)	Adequate but unevenly distributed across scaffold-plus-membrane combinations	Identifiable	Not serious	Serious: global network inconsistency	Some concern: model checks were performed at network level rather than component-specific level	Serious: contributing evidence included non-low risk-of-bias judgments	Low

Allograft	-0.77 (-1.16 to -0.38)	Adequate but unevenly distributed; appeared as standalone allograft and in PRF or membrane combinations	Identifiable	Not serious	Serious: global network inconsistency	Some concern: model checks were performed at network level rather than component-specific level	Serious: contributing evidence included non-low risk-of-bias judgments	Low
Xenograft	-0.57 (-1.20 to 0.06)	Sparse and unevenly distributed, with limited standalone and combined-component contrasts	Identifiable	Serious	Serious: global network inconsistency	Some concern: model checks were performed at network level rather than component-specific level	Serious: contributing evidence included non-low risk-of-bias judgments	Very low
Collagen scaffold	-0.42 (-1.07 to 0.23)	Sparse across component combinations; evidence mainly from collagen-based scaffold or socket-coverage contrasts	Identifiable	Serious	Serious: global network inconsistency	Some concern: model checks were performed at network level rather than component-specific level	Serious: contributing evidence included non-low risk-of-bias judgments	Very low
PRF adjunct	-0.40 (-0.66 to -0.14)	Adequate but unevenly distributed; appeared alone and in scaffold-containing combinations	Identifiable	Not serious	Serious: global network inconsistency	Some concern: model checks were performed at network level rather than component-specific level	Serious: contributing evidence included non-low risk-of-bias judgments	Low
Alloplast	0.01 (-0.51 to 0.53)	Limited and unevenly distributed across alloplast-based contrasts	Identifiable	Serious	Serious: global network inconsistency	Some concern: model checks were performed at network level rather than component-specific level	Serious: contributing evidence included non-low risk-of-bias judgments	Very low

Table S18. Histomorphometric outcomes reported in included studies

Study	Follow-up	New bone / histomorphometry reported	Residual graft / non-mineralized tissue reported	Method	Outcomes
Estrin et al. (2026) [4]	3 months; wound healing at 2 weeks	No/unclear	No/unclear	NR	CBCT ridge width/height, soft tissue thickness, early wound healing
Alrayyes et al. (2023) [5]	6 months	Yes	No/unclear	Histology/histomorphometry	CBCT ridge height/width; histology at implant placement
Talebi Ardakani et al. (2024) [6]	3 months	Yes	No/unclear	Histology/histomorphometry	CBCT ridge dimensions; clinical ridge width; histomorphometry
Gowda et al. (2023) [7]	4 months	No/unclear	No/unclear	NR	Clinical and CBCT ridge height/width
Zampara et al. (2022) [8]	NR in extracted text; biopsy at implant re-entry	Yes	No/unclear	Histology/histomorphometry	Clinical healing; histologic/histomorphometric outcomes

Gokul et al. (2025) [9]	6 months	No/unclear	No/unclear	NR	Clinical and CBCT/radiographic ridge parameters
Elfana et al. (2021) [10]	4 months	Yes	No/unclear	Histology/histomorphometry	CBCT dimensional change; histologic healing
Abad et al. (2023) [11]	4 months	No/unclear	No/unclear	NR	CBCT ridge dimensions, STL volumetric contour, patient-reported outcomes
Hussain et al. (2023) [12]	NR in extracted table; histology/radiography at re-entry	Yes	No/unclear	Histology/histomorphometry	Clinical, tomographic/radiographic and histologic ARP outcomes
Clark et al. (2018) [13]	NR in extracted summary; biopsy at re-entry	Yes	No/unclear	Histology/histomorphometry	Histomorphometry, micro-CT bone density and ridge measurements
Thakkar et al. (2016) [14]	NR in extracted summary	No/unclear	No/unclear	NR	Clinical and radiographic bone fill/ridge dimensions
Schnutenhaus et al. (2018) [15]	8-11 weeks	No/unclear	No/unclear	NR	CBCT/radiographic ridge preservation; clinical outcomes
Ouyyamwongs et al. (2019) [16]	2, 4, 6 and 8 weeks; main endpoint 8 weeks	No/unclear	No/unclear	NR	Clinical/radiographic ridge width and height
Dhamija et al. (2020) [17]	6 months; implant placement around 7 months	Yes	No/unclear	Histology/histomorphometry	Clinical, histologic and radiologic bone formation
Girish Kumar et al. (2018) [18]	6 months; broader follow-up to 18 months reported	No/unclear	No/unclear	NR	Clinical and radiographic socket healing/ridge preservation
Ivanova et al. (2019) [19]	4 months	Yes	No/unclear	Histology/histomorphometry	Dimensional ridge alteration; histomorphometry
Castro et al. (2021) [20]	3 months	No/unclear	No/unclear	NR	CBCT/radiographic dimensional change and bone structure
Demetter et al. (2017) [21]	18-20 weeks	Yes	Yes	Histology/histomorphometry	Histomorphometric vital bone/residual graft; clinical ridge dimensions
Corning and Mealey (2019) [22]	12 weeks	Yes	No/unclear	Histology/histomorphometry	Clinical ridge changes and primary histologic outcomes
Saito et al. (2021) [23]	16-20 weeks; 12-month implant follow-up reported	Yes	No/unclear	Histology/histomorphometry	CBCT ridge dimensions; histology; implant-related outcomes
Bajaj et al. (2025) [24]	3-4 months/16 weeks	No/unclear	No/unclear	NR	Clinical and radiographic ridge dimensions
Shahdad et al. (2020) [25]	6 months/22 weeks	No/unclear	No/unclear	NR	CBCT/radiographic ridge preservation; clinical outcomes
Choi et al. (2025) [26]	5 months	Yes	No/unclear	Histology/histomorphometry	Radiologic and histologic ARP outcomes
Nainoor et al. (2026) [27]	4 months	No/unclear	No/unclear	NR	Clinical and radiographic ridge dimensions
Barone et al. (2008) [28]	7 months	Yes	No/unclear	Histology/histomorphometry	Clinical ridge dimensions; histomorphometry
Nunes et al. (2018) [29]	6 months	No/unclear	No/unclear	NR	Clinical and cone-beam tomographic ridge dimensions
Azangookhiavi et al. (2020) [30]	12 weeks/3 months	No/unclear	No/unclear	NR	Clinical and radiographic ridge height/width
Schnutenhaus et al. (2020) [31]	8 - 1 weeks CBCT; 11 - 1 weeks implant placement	Yes	No/unclear	Histology/histomorphometry	CBCT bone density; histomorphometry; implant primary stability
Aladmawy et al. (2022) [32]	6 months	Yes	Yes	Histology/histomorphometry	Histomorphometric new vital bone/residual graft/marrow tissue
Wr-bel et al. (2026) [33]	7-10 days and 6 months	Yes	No/unclear	Histology/histomorphometry	Micro-CBCT ridge dimensions/bone density; histology
Barone et al. (2016) [34]	1 and 3 months	No/unclear	No/unclear	NR	Scanned plaster casts/volumetric ridge remodeling; CBCT-based VOI described

Table S19. Patient-reported, wound-healing and safety outcomes reported in included studies

Study	Follow-up	Patient-reported outcomes	Wound/soft-tissue healing	Adverse events/safety	Outcomes

Estrin et al. (2026) [4]	3 months; wound healing at 2 weeks	Not clearly reported	Reported	Not clearly reported	CBCT ridge width/height, soft tissue thickness, early wound healing
Alrayyes et al. (2023) [5]	6 months	Not clearly reported	Not clearly reported	Not clearly reported	CBCT ridge height/width; histology at implant placement
Talebi Ardakani et al. (2024) [6]	3 months	Not clearly reported	Not clearly reported	Not clearly reported	CBCT ridge dimensions; clinical ridge width; histomorphometry
Gowda et al. (2023) [7]	4 months	Not clearly reported	Not clearly reported	Not clearly reported	Clinical and CBCT ridge height/width
Zampara et al. (2022) [8]	NR in extracted text; biopsy at implant re-entry	Not clearly reported	Reported	Not clearly reported	Clinical healing; histologic/histomorphometric outcomes
Gokul et al. (2025) [9]	6 months	Not clearly reported	Not clearly reported	Not clearly reported	Clinical and CBCT/radiographic ridge parameters
Elfana et al. (2021) [10]	4 months	Not clearly reported	Reported	Not clearly reported	CBCT dimensional change; histologic healing
Abad et al. (2023) [11]	4 months	Reported	Not clearly reported	Not clearly reported	CBCT ridge dimensions, STL volumetric contour, patient-reported outcomes
Hussain et al. (2023) [12]	NR in extracted table; histology/radiography at re-entry	Not clearly reported	Not clearly reported	Not clearly reported	Clinical, tomographic/radiographic and histologic ARP outcomes
Clark et al. (2018) [13]	NR in extracted summary; biopsy at re-entry	Not clearly reported	Not clearly reported	Not clearly reported	Histomorphometry, micro-CT bone density and ridge measurements
Thakkar et al. (2016) [14]	NR in extracted summary	Not clearly reported	Not clearly reported	Not clearly reported	Clinical and radiographic bone fill/ridge dimensions
Schnutenhaus et al. (2018) [15]	8-11 weeks	Not clearly reported	Not clearly reported	Not clearly reported	CBCT/radiographic ridge preservation; clinical outcomes
Ouyyamwongs et al. (2019) [16]	2, 4, 6 and 8 weeks; main endpoint 8 weeks	Not clearly reported	Not clearly reported	Not clearly reported	Clinical/radiographic ridge width and height
Dhamija et al. (2020) [17]	6 months; implant placement around 7 months	Not clearly reported	Not clearly reported	Not clearly reported	Clinical, histologic and radiologic bone formation
Girish Kumar et al. (2018) [18]	6 months; broader follow-up to 18 months reported	Not clearly reported	Reported	Not clearly reported	Clinical and radiographic socket healing/ridge preservation
Ivanova et al. (2019) [19]	4 months	Not clearly reported	Not clearly reported	Not clearly reported	Dimensional ridge alteration; histomorphometry
Castro et al. (2021) [20]	3 months	Not clearly reported	Not clearly reported	Not clearly reported	CBCT/radiographic dimensional change and bone structure
Demetter et al. (2017) [21]	18-20 weeks	Not clearly reported	Not clearly reported	Not clearly reported	Histomorphometric vital bone/residual graft; clinical ridge dimensions
Corning and Mealey (2019) [22]	12 weeks	Not clearly reported	Not clearly reported	Not clearly reported	Clinical ridge changes and primary histologic outcomes
Saito et al. (2021) [23]	16-20 weeks; 12-month implant follow-up reported	Not clearly reported	Not clearly reported	Not clearly reported	CBCT ridge dimensions; histology; implant-related outcomes
Bajaj et al. (2025) [24]	3-4 months/16 weeks	Not clearly reported	Not clearly reported	Not clearly reported	Clinical and radiographic ridge dimensions
Shahdad et al. (2020) [25]	6 months/22 weeks	Not clearly reported	Not clearly reported	Not clearly reported	CBCT/radiographic ridge preservation; clinical outcomes
Choi et al. (2025) [26]	5 months	Not clearly reported	Not clearly reported	Not clearly reported	Radiologic and histologic ARP outcomes
Nainoor et al. (2026) [27]	4 months	Not clearly reported	Not clearly reported	Not clearly reported	Clinical and radiographic ridge dimensions
Barone et al. (2008) [28]	7 months	Not clearly reported	Not clearly reported	Not clearly reported	Clinical ridge dimensions; histomorphometry
Nunes et al. (2018) [29]	6 months	Not clearly reported	Not clearly reported	Not clearly reported	Clinical and cone-beam tomographic ridge dimensions
Azangookhiavi et al. (2020) [30]	12 weeks/3 months	Not clearly reported	Not clearly reported	Not clearly reported	Clinical and radiographic ridge height/width
Schnutenhaus et al. (2020) [31]	8 - 1 weeks CBCT; 11 - 1 weeks implant placement	Not clearly reported	Not clearly reported	Not clearly reported	CBCT bone density; histomorphometry; implant primary stability
Aladmawy et al. (2022) [32]	6 months	Not clearly reported	Not clearly reported	Not clearly reported	Histomorphometric new vital bone/residual graft/marrow tissue
Wr-bel et al. (2026) [33]	7-10 days and 6 months	Not clearly reported	Not clearly reported	Not clearly reported	Micro-CBCT ridge dimensions/bone density;

					histology
Barone et al. (2016) [34]	1 and 3 months	Not clearly reported	Not clearly reported	Not clearly reported	Scanned plaster casts/volumetric ridge remodeling; CBCT-based VOI described

Table S20. Arm-level primary outcome data transformations and statistical assumptions

study_id	study	arm	treatment_node	outcome	measurement_level	original_mean	original_sd	n	harmonized_loss_mean	follow_up	measurement_method	directly_meta_usable	direction_handling	notes
1	Estrin et al., 2026 [4]	Collagen	COL	horizontal width change	1 mm below crest	1.31	0.74	11	1.31	3 months	CBCT	Yes	loss_positive	NR
1	Estrin et al., 2026 [4]	Collagen	COL	buccal vertical height change	buccal height	1.17	0.53	11	1.17	3 months	CBCT	Yes	loss_positive	NR
1	Estrin et al., 2026 [4]	Membrane	PRF-M	horizontal width change	1 mm below crest	1.29	0.88	11	1.29	3 months	CBCT	Yes	loss_positive	NR
1	Estrin et al., 2026 [4]	Membrane	PRF-M	buccal vertical height change	buccal height	1.15	0.53	11	1.15	3 months	CBCT	Yes	loss_positive	NR
1	Estrin et al., 2026 [4]	Membrane w/solid	PRF-M	horizontal width change	1 mm below crest	0.99	0.41	11	0.99	3 months	CBCT	Yes	loss_positive	NR
1	Estrin et al., 2026 [4]	Membrane w/solid	PRF-M	buccal vertical height chan	buccal height	0.81	0.51	11	0.81	3 months	CBCT	Yes	loss_positive	NR

				ge										
1	Estrin et al., 2026 [4]	Bio-Filler	PRF	horizontal width change	1 mm below crest	1.4	0.66	11	1.4	3 months	CBCT	Yes	loss_positive	NR
1	Estrin et al., 2026 [4]	Bio-Filler	PRF	buccal vertical height change	buccal height	1.2	0.64	11	1.2	3 months	CBCT	Yes	loss_positive	NR
1	Estrin et al., 2026 [4]	Bio-Filler w/solid	PRF	horizontal width change	1 mm below crest	1.27	0.42	11	1.27	3 months	CBCT	Yes	loss_positive	NR
1	Estrin et al., 2026 [4]	Bio-Filler w/solid	PRF	buccal vertical height change	buccal height	1.19	0.93	11	1.19	3 months	CBCT	Yes	loss_positive	NR
3	Talebi Ardakani et al., 2024 [6]	Control: allograft + collagen	ALG+COL	horizontal width change	radiographic bone width reduction	1.07	0.4	6	1.07	4 months	CBCT/radiographic	Yes	loss_positive	NR
3	Talebi Ardakani et al., 2024 [6]	Control: allograft + collagen	ALG+COL	buccal vertical height change	radiographic bone height reduction	1.2	0.46	6	1.2	4 months	CBCT/radiographic	Yes	loss_positive	NR
3	Talebi Ardakani	Allograft + collagen + I-PRF	ALG+PRF+COL	horizontal width	radiographic bone width reduction	0.67	0.39	6	0.67	4 months	CBCT/radiographic	Yes	loss_positive	NR

	et al., 2024 [6]			change												
3	Talebi Ardakani et al., 2024 [6]	Allograft + collagen + I-PRF	ALG+PRF+COL	buccal vertical height change	radiographic bone height reduction	0.96	0.34	6	0.96		4 months	CBCT/radiographic	Yes		loss_positive	NR
4	Gowda et al., 2023 [7]	PDDM block + A-PRF/i-PRF	DT+PRF	horizontal width change	radiographic horizontal width at crest	0.29	0.1	8	0.29		4 months	CBCT	Yes		loss_positive	Reported loss; test group baseline-follow-up direction should be verified.
4	Gowda et al., 2023 [7]	PDDM block + A-PRF/i-PRF	DT+PRF	buccal vertical height change	radiographic buccal cortical height	0.24	0.09	8	0.24		4 months	CBCT	Yes		loss_positive	NR
4	Gowda et al., 2023 [7]	A-PRF+ plug alone	PRF	horizontal width change	radiographic horizontal width at crest	1.45	0.02	8	1.45		4 months	CBCT	Yes		loss_positive	Reported loss; test group baseline-fol

														low-up direction should be verified.
4	Gowda et al., 2023 [7]	A-PRF+ plug alone	PRF	buccal vertical height change	radiographic buccal cortical height	2.23	0.05	8	2.23	4 months	CBCT	Yes	loss_positive	NR
7	Elfana et al., 2021 [10]	AWTG	DT	horizontal width change	buccolingual ridge width loss	0.85	0.38	10	0.85	6 months	clinical/CBCT	Yes	loss_positive	NR
7	Elfana et al., 2021 [10]	AWTG	DT	buccal vertical height change	buccal ridge height loss	0.61	0.2	10	0.61	6 months	clinical/CBCT	Yes	loss_positive	NR
7	Elfana et al., 2021 [10]	ADDG	DT	horizontal width change	buccolingual ridge width loss	1.02	0.45	10	1.02	6 months	clinical/CBCT	Yes	loss_positive	NR
7	Elfana et al., 2021 [10]	ADDG	DT	buccal vertical height change	buccal ridge height loss	0.72	0.27	10	0.72	6 months	clinical/CBCT	Yes	loss_positive	NR
8	Abad et al., 2023 [1	Natural healing	NH	horizontal width chan	HRW 1 mm	-6.67	3.13	13	6.67	4 months	CBCT	Yes	negative_loss	NR

	1]			ge										
8	Abad et al., 2023 [11]	Natural healing	NH	buccal vertical height change	RED-V	2.73	2.19	13	2.73	4 months	CBCT	Yes	loss_positive	NR
8	Abad et al., 2023 [11]	L-PRF	PRF	horizontal width change	HRW 1 mm	-6.37	2.95	14	6.37	4 months	CBCT	Yes	negative_loss	NR
8	Abad et al., 2023 [11]	L-PRF	PRF	buccal vertical height change	RED-V	1.91	1.43	14	1.91	4 months	CBCT	Yes	loss_positive	NR
9	Hussain et al., 2023 [12]	Autogenous dentin biomaterial	DT	horizontal width change	ridge width at 2 mm	-1.47	1.22	14	1.47	6 months	CBCT	Yes	negative_loss	NR
9	Hussain et al., 2023 [12]	Natural healing	NH	horizontal width change	ridge width at 2 mm	-3.54	1.26	15	3.54	6 months	CBCT	Yes	negative_loss	NR
10	Clark et al., 2018 [13]	Blood clot	NH	horizontal width change	coronal ridge width loss	2.9	1.7	10	2.9	3 months	clinical/CBCT	Yes	loss_positive	NR
10	Clark et al., 2018 [13]	Blood clot	NH	buccal vertical height change	ridge height loss	3.8	2	10	3.8	3 months	clinical/CBCT	Yes	loss_positive	NR
10	Cl	A-	PRF	ho	coronal	2.8	1.2	1	2.8	3	clinical/	Yes	loss_	N

	ark et al., 2018 [13]	PRF		rizontal width change	ridge width loss			0		months	CBCT		positive	R
10	Clark et al., 2018 [13]	A-PRF	PRF	buccal vertical height change	ridge height loss	1.8	2.1	10	1.8	3 months	clinical/CBCT	Yes	loss_positive	NR
10	Clark et al., 2018 [13]	A-PRF+FDBA	ALG+PRF	horizontal width change	coronal ridge width loss	1.9	1.1	10	1.9	3 months	clinical/CBCT	Yes	loss_positive	NR
10	Clark et al., 2018 [13]	A-PRF+FDBA	ALG+PRF	buccal vertical height change	ridge height loss	1	2.3	10	1	3 months	clinical/CBCT	Yes	loss_positive	NR
10	Clark et al., 2018 [13]	FDBA	ALG	horizontal width change	coronal ridge width loss	2.5	1.1	10	2.5	3 months	clinical/CBCT	Yes	loss_positive	NR
10	Clark et al., 2018 [13]	FDBA	ALG	buccal vertical height change	ridge height loss	2.2	1.8	10	2.2	3 months	clinical/CBCT	Yes	loss_positive	NR
11	Thakkar et al., 2016 [14]	DFDBA	ALG	horizontal width change	ridge width loss	1.36	0.703	18	1.36	180 days	clinical	Yes	loss_positive	NR
11	Thakkar	DFDBA	ALG	buccal ve	ridge height change	-1.39	0.502	18	1.39	180 days	clinical	Yes	negative_loss	NR

	et al., 2016 [14]			rtical height change										
11	Thakkar et al., 2016 [14]	DFDBA + PRF	ALG+PRF	horizontal width change	ridge width loss	0.75	0.493	18	0.75	180 days	clinical	Yes	loss_positive	NR
11	Thakkar et al., 2016 [14]	DFDBA + PRF	ALG+PRF	buccal vertical height change	ridge height change	-1.08	0.429	18	1.08	180 days	clinical	Yes	negative_loss	NR

References

[1] CLARK D, PAYDAR S, RAJENDRAN Y, et al. Platelet-rich fibrin for ridge preservation: a randomized controlled clinical study[J]. Journal of dental research, 2016.

[2] ROLOTTI D, CHIOU L L, FREILICH M, et al. A Pilot Study Comparing Dimensional Alterations After Alveolar Ridge Preservation with Xenograft Versus Allograft[J]. International Journal of Periodontics & Restorative Dentistry, 2025: 1-25.

[3] IBRAHIM T, PRAKASH P, APPUKUTAN D, et al. Comparative Evaluation of Bone Dimensional Changes in Extraction Sockets Preserved With Calcium Phosphosilicate Bone Substitutes With and Without Platelet-Rich Fibrin: A Randomized Controlled Clinical Trial[J]. The International Journal of Periodontics & Restorative Dentistry, 2022, 42(2): 261-267.

[4] ESTRIN N E, AHMAD P, TRAN T B, et al. The Evolution of Extended Platelet-Rich Fibrin Membranes for Socket Grafting: Part Two: A Randomized Clinical Trial Comparing These Membranes with Collagen Membranes[J]. Dentistry Journal, 2026, 14(1): 45.

[5] ALRAYYES Y, ALORAINI S, ALSHAGROUD R, et al. Extraction and Socket Preservation before Implant Placement Using Freeze-Dried Allograft (FDBA) and Platelet-Rich Fibrin in Smokers: Radiographic and Histological Evaluation[J]. Applied Sciences, 2023, 13(12): 7076.

[6] TALEBI ARDAKANI M R, REZAEI ESFAHROOD Z, MASHHADIABBAS F, et al. Comparison of Histological, Clinical, and Radiographic Outcomes of Postextraction Ridge Preservation by Allogenic Bone Grafting With and Without Injectable Platelet-Rich Fibrin: A Double-Blinded Randomized Controlled Clinical Trial[J]. International Journal of Dentistry, 2024, 2024(1): 8850664.

[7] GOWDA T M, JAYASHRI M, VENKATESH U G, et al. Autologous tooth bone graft block compared with advanced platelet-rich fibrin in alveolar ridge preservation: A clinico-radiographic study[J]. Journal of Indian Society of Periodontology, 2023, 27(6): 619-625.

[8] ZAMPARA E, ALSHAMMARI M, DE BORTOLI J, et al. A Histologic and Histomorphometric Evaluation of an Allograft, Xenograft, and Alloplast Graft for Alveolar Ridge Preservation in Humans: A Randomized Controlled Clinical Trial[J]. Journal of Oral Implantology, 2022, 48(6): 541-549.

[9] MOPATI N R G, RAMESH K S, PENMETSA S G, et al. A comparative evaluation of eggshell derived hydroxyapatite and demineralized freeze-dried bone allograft in alveolar ridge preservation – a randomized clinical trial[J]. Biomaterial Investigations in Dentistry, 2025, 12: 175-183.

[10] ELFANA A, EL-KHOLY S, SALEH H A, et al. Alveolar ridge preservation using autogenous whole-tooth versus demineralized dentin grafts: A randomized controlled clinical trial[J]. Clinical Oral Implants Research, 2021, 32(5): 539-548.

[11] ABAD C E, SANZ-SANCHEZ I, SERRANO V, et al. Efficacy of the application of leukocyte and platelet-rich fibrin (L-PRF) on alveolar ridge preservation. A randomized controlled clinical trial[J]. Clinical Implant Dentistry and Related Research, 2023, 25(3): 592-604.

[12] HUSSAIN A A, AL-QUISI A F, ABDULKAREEM A A. Efficacy of Autogenous Dentin Biomaterial on Alveolar Ridge Preservation: A Randomized Controlled Clinical Trial[J]. BioMed Research International, 2023, 2023(1): 7932432.

[13] CLARK D, RAJENDRAN Y, PAYDAR S, et al. Advanced platelet-rich fibrin and freeze-dried bone allograft for ridge preservation: A randomized controlled clinical trial[J]. Journal of Periodontology, 2018, 89(4): 379-387.

[14] THAKKAR D, DESHPANDE N, DAVE D, et al. A comparative evaluation of extraction socket preservation with demineralized freeze-dried bone allograft alone and along with platelet-rich fibrin: A clinical and radiographic study[J]. Contemporary Clinical Dentistry, 2016, 7(3): 371.

[15] SCHNUTENHAUS S, DOERING I, DREYHAUPT J, et al. Alveolar ridge preservation with a collagen material: a randomized controlled trial[J]. Journal of Periodontal & Implant Science, 2018, 48(4): 236.

[16] OUYYAMWONGS W, LEEPONG N, SUTTAPREYASRI S. Alveolar Ridge Preservation Using Autologous Demineralized Tooth Matrix and Platelet-Rich Fibrin Versus Platelet-Rich Fibrin Alone[J]. Implant Dentistry, 2019, 28(5): 455-462.

[17] DHAMIJA R, SHETTY V, VINEETH K, et al. Socket preservation with demineralized freeze-dried bone allograft and platelet-rich fibrin for implant site development: A randomized controlled trial[J]. The Journal of Indian Prosthodontic Society, 2020, 20(3): 304.

[18] GIRISH KUMAR N, CHAUDHARY R, KUMAR I, et al. To assess the efficacy of socket plug technique using platelet rich fibrin with or without the use of bone substitute in alveolar ridge

preservation: a prospective randomised controlled study[J]. Oral and Maxillofacial Surgery, 2018, 22(2): 135-142.

[19] IVANOVA V, CHENCHEV I, ZLATEV S, et al. DIMENSIONAL RIDGE ALTERATIONS AND HISTOMORPHOMETRIC ANALYSIS FOLLOWING SOCKET PRESERVATION WITH PRF OR ALLOGRAFT. RANDOMIZED CONTROLLED CLINICAL STUDY[J]. Journal of IMAB - Annual Proceeding (Scientific Papers), 2019, 25(4): 2853-2861.

[20] CASTRO A B, VAN DESSEL J, TEMMERMAN A, et al. Effect of different platelet-rich fibrin matrices for ridge preservation in multiple tooth extractions: A split-mouth randomized controlled clinical trial[J]. Journal of Clinical Periodontology, 2021, 48(7): 984-995.

[21] DEMETTER R S, CALAHAN B G, MEALEY B L. Histologic Evaluation of Wound Healing After Ridge Preservation With Cortical, Cancellous, and Combined Cortico-Cancellous Freeze-Dried Bone Allograft: A Randomized Controlled Clinical Trial[J]. Journal of Periodontology, 2017, 88(9): 860-868.

[22] CORNING P J, MEALEY B L. Ridge preservation following tooth extraction using mineralized freeze-dried bone allograft compared to mineralized solvent-dehydrated bone allograft: A randomized controlled clinical trial[J]. Journal of Periodontology, 2019, 90(2): 126-133.

[23] SAITO H, COUSO-QUEIRUGA E, SHIAU H J, et al. Evaluation of poly lactic-co-glycolic acid-coated β-tricalcium phosphate for alveolar ridge preservation: A multicenter randomized controlled trial[J]. Journal of Periodontology, 2021, 92(4): 524-535.

[24] BAJAJ P, DHADSE P V, PATIL R T, et al. Evaluation of Effectiveness of Nanocrystalline Hydroxyapatite and Demineralized Bone Matrix Combined with Titanium-platelet Rich Fibrin for Ridge Preservation: A Randomized Controlled Clinical Trial[J]. The Journal of Contemporary Dental Practice, 2025, 25(11): 1069-1076.

[25] SHAHDAD S, GAMBLE E, MATANI J, et al. Randomized clinical trial comparing PEG-based synthetic to porcine-derived collagen membrane in the preservation of alveolar bone following tooth extraction in anterior maxilla[J]. Clinical Oral Implants Research, 2020, 31(10): 1010-1024.

[26] CHOI Y, CHEONG J, SONG Y, et al. Moldable and Particulate Bone Material in Alveolar Ridge Preservation: A Multicenter Randomized Controlled Trial[J]. Clinical Implant Dentistry and Related Research, 2025, 27(2): e70011.

[27] NAINOOR N, SHOBHA E S, PRASHANTH N T, et al. Comparison of Clinical and Radiographic Assessment of Efficacy of Eggshell-Derived Nanohydroxyapatite (EnHA) and Demineralized Freeze-Dried Bone Allograft (DFDBA), Both Covered with Platelet-Rich Fibrin (PRF) as a Barrier Membrane in Socket Preservation: A Randomized Controlled Clinical Trial[J]. Journal of Maxillofacial and Oral Surgery, 2026, 25(1): 225-237.

[28] BARONE A, ALDINI N N, FINI M, et al. Xenograft Versus Extraction Alone for Ridge Preservation After Tooth Removal: A Clinical and Histomorphometric Study[J]. Journal of Periodontology, 2008, 79(8): 1370-1377.

[29] NUNES F A S, PIGNATON T B, NOVAES A B, et al. Evaluation of a bone substitute covered with a collagen membrane for ridge preservation after tooth extraction. Clinical and tomographic randomized controlled study in humans[J]. Clinical Oral Implants Research, 2018, 29(4): 424-433.

[30] AZANGOOKHIAVI H, GHODSI S, JALIL F, et al. Comparison of the Efficacy of Platelet-Rich Fibrin and Bone Allograft for Alveolar Ridge Preservation after Tooth Extraction: A Clinical Trial[J]. Frontiers in Dentistry, 2020, 17(1): 1-6.

[31] SCHNUTENHAUS S, GÖTZ W, DREYHAUPT J, et al. Associations among Primary Stability, Histomorphometric Findings, and Bone Density: A Prospective Randomized Study after Alveolar Ridge Preservation with a Collagen Cone[J]. Dentistry Journal, 2020, 8(4): 112.

[32] ALADMAWY M A, NATTO Z S, KREITZER M, et al. Histological and histomorphometric evaluation of alveolar ridge preservation using an allograft and nonresorbable membrane with and without primary closure: A pilot randomized controlled clinical trial[J]. Medicine, 2022, 101(26): e29769.

[33] WRÓBEL P, JĘDZIEROWSKA M, PIECUCH A, et al. Evaluation of the Effectiveness of the Socket Preservation Technique Using Allogeneic and Xenogeneic Materials: A Randomized Controlled Trial[J]. Journal of Functional Biomaterials, 2026, 17(6): 262.

[34] BARONE A, TOTI P, QUARANTA A, et al. Volumetric analysis of remodelling pattern after ridge preservation comparing use of two types of xenografts. A multicentre randomized clinical trial[J]. Clinical Oral Implants Research, 2016, 27(11): e105-e115.
